# Genomic architecture of non-nominal offspring risk in a simulated composite cattle breed

**DOI:** 10.1186/s12711-026-01093-2

**Published:** 2026-10-16

**Authors:** Miguel Gallach

**Affiliations:** GC Genomics, C. Exposición 6, Mairena del Aljarafe, Seville 41927 Spain

## Abstract

**Background:**

Composite cattle breeds are designed to combine complementary attributes from different founder breeds while retaining part of the heterosis generated by crossbreeding. Brangus cattle are nominally defined as 5/8 Angus and 3/8 Brahman, but the same global breed composition can be produced by different genomic architectures. This distinction may be important because animals with the same Brahman ancestry proportion may differ in ancestry heterozygosity, homozygous ancestry composition and segregation behaviour when used as parents. The objective of this study was to use controlled simulations to evaluate whether genomic architecture affects the risk of producing offspring outside the nominal Brangus ancestry range.

**Results:**

We simulated two alternative pathways to the same expected Brahman ancestry proportion of 0.375. Although both pathways produced similar mean Brahman ancestry, the direct pathway generated a broader ancestry distribution than the indirect pathway, with a higher proportion of individuals outside the nominal interval. After filtering candidate sires to the nominal interval, formation-pathway-dependent genomic architectures remained evident. In a mixed *inter se* mating analysis extended to 100 generations and three population-size scenarios, pathway-specific ancestry-state differences were progressively eroded and the standard deviation of Brahman ancestry proportion declined after an early maximum, but non-nominal frequencies remained non-negligible over the simulated time horizon. Family-level simulations showed that matings between nominal parents differed substantially in their probability of producing non-nominal daughters. This risk depended on both the distance between the expected family mean and the target ancestry and the block-weighted segregation potential of the parental genomes. An exploratory quasibinomial model supported positive associations between non-nominal risk and displacement from the target and high-variance parental block structures. Finally, a heuristic-assisted simulation-informed sire allocation strategy increased the proportion of nominal daughters relative to random sire allocation, with the greatest gains obtained from broader sire catalogues.

**Conclusions:**

Nominal breed composition does not define a unique genomic state. In simulated Brangus cattle, animals that were equivalent in global ancestry could differ in genomic architecture and in the offspring ancestry distributions generated by specific matings. These results suggest that composite-breed management should consider not only the ancestry proportion of individual animals, but also the expected outcome of sire–dam pairs.

**Supplementary Information:**

The online version contains supplementary material available at https://doi.org/10.1186/s12711-026-01093-2.

## Background

Composite cattle breeds have been developed to combine favourable attributes from different founder breeds with retained heterosis within a single breeding population [1, 2]. In contrast to terminal or rotational crossbreeding systems, a composite breed is intended to be maintained as a breeding population after formation, allowing selection to proceed within the composite while retaining part of the heterosis generated by the founder-breed crosses [1–3]. This approach is particularly relevant in production systems in which breed complementarity is needed to balance adaptation, reproduction, maternal performance, growth and product quality [3–5].

A central rationale for crossbreeding is the simultaneous use of additive breed differences and non-additive heterotic effects. Gregory and Cundiff described beef cattle crossbreeding systems as a way to use both components at the same time: additive breed effects allow genetic resources to be matched to the production environment and market requirements, whereas heterosis improves performance through non-additive genetic effects [3]. This distinction is important because the advantage of a crossbred or composite animal may arise partly from additive breed complementarity and partly from true heterosis. In tropical and subtropical systems, this distinction is especially relevant because *Bos indicus*-derived breeds such as Brahman contribute adaptation to heat, parasites, nutritional stress and other environmental constraints, whereas *Bos taurus* breeds often contribute higher productivity, carcass quality or dairy potential [4–6].

Crossbreeding between *Bos taurus* and *Bos indicus* populations has therefore been widely used to combine adaptation and production. Koger emphasized that Brahman–European crosses generally express high levels of heterosis and that several Zebu-derived *inter se* mating groups or composite breeds arose from this context, including Santa Gertrudis, Beefmaster, Brangus, Braford, Barzona and Charbray [5]. Composite breeds provide a practical alternative to continuous crossbreeding because, once formed, they can be managed more like straightbred populations while still retaining part of the heterozygosity and heterosis generated during breed formation [1, 3]. This is particularly important for smaller herds, where complex rotational or terminal crossbreeding systems can be difficult to implement [1, 3].

Several cattle composite breeds have been developed around defined breed-composition targets. A common structure in Zebu–European composites is an intermediate breed-composition target, often involving approximately 5/8 contribution from one breed or breed group and 3/8 from another. Brangus, for example, is classically defined as 5/8 Angus (A) and 3/8 Brahman (B), corresponding to a target Brahman ancestry proportion of 0.375. Similar intermediate targets have been used in other composites, including Braford and Santa Gertrudis, and dairy composite systems such as Girolando include Holstein × Gyr breed-composition classes such as the 5/8 Holstein and 3/8 Gyr type. These intermediate compositions provide a practical compromise between productivity and adaptation, but they do not necessarily maximize retained heterozygosity. Koger noted that increasing the number of contributing breeds and using more balanced breed proportions can increase residual breed heterozygosity, and that this point had been overlooked in the promotion of 3/8 + 5/8 Zebu–European composites [5].

Genomic tools have made it possible to estimate breed composition directly from marker data and to describe how founder-breed ancestries are distributed across the genome. In Brangus, genomic data have been used to estimate population structure and genomic breed composition [7]. Paim et al. showed that genetic drift, selection and complementarity can shift the genomic architecture of composite cattle populations away from expected founder-breed proportions [8], and later showed that selection signatures may differ in their genomic breed composition [9]. More broadly, Hay et al. characterized the genetic architecture of a composite beef cattle population and showed how genomic data can be used to describe the breed composition and structure of composite populations [10]. Li et al. estimated genomic breed composition in 3605 Brangus cattle and reported an average of 70.2% Angus and 29.8% Brahman, with non-Ultrablack Brangus averaging 67.4% Angus and 32.6% Brahman [11]. In Argentine Brangus, Álvarez Cecco et al. reported an average Brahman composition of approximately 35%, with substantial individual variation [12]. Together, these studies show that realized genomic composition in Brangus can deviate from the nominal 3/8 Brahman expectation and that breed composition is a variable genomic property rather than a fixed label.

Beyond Brangus, studies of other Zebu–European synthetic or composite breeds support the broader relevance of genomic breed composition and local ancestry as descriptors of composite populations. Genomic breed composition has been estimated in Santa Gertrudis as well as Brangus, showing that realized genomic ancestry can be compared with pedigree expectations in composite beef populations [13]. Local taurine and indicine haplotype representation has also been analysed across Brangus, Beefmaster and Santa Gertrudis, demonstrating that chromosomal regions can differ from expected global ancestry proportions within and across breeds [14]. Braford populations have likewise been studied from the perspective of population structure and genetic diversity during breed introduction and expansion [15]. In dairy cattle, Girolando provides a prominent Holstein × Gyr composite system in which defined breed-composition classes, including the 5/8 Holstein and 3/8 Gyr type, are used to balance milk production and tropical adaptation [16]. Studies in Girolando have evaluated genetic parameters, breed-group effects and heat-stress tolerance across Holstein–Gyr genetic groups [17], and genomic work using high-density markers has estimated average Girolando ancestry at approximately 0.66 Holstein and 0.34 Gyr, close to the 5/8 + 3/8 target [18]. Together, these studies show that breed composition, local ancestry, diversity and adaptation have become central descriptors of Zebu–European composite breeds, but they still mainly characterize ancestry at the level of individuals, populations, breed classes or genomic regions rather than at the level of expected offspring distributions from specific sire–dam pairs.

Genomic breed composition has also been connected to heterosis-related quantities in crossbred cattle. Akanno et al. estimated breed fractions from genotyping array data in Angus, Charolais and Hereford crosses and used these fractions to calculate retained heterozygosity and retained heterosis expectations [19]. They reported high correlations between pedigree- and genome-based breed composition estimates, and a strong correlation between pedigree-based retained heterosis and genome-based retained heterozygosity. These results support the use of genomic data to estimate breed composition and heterosis-related expectations in crossbred populations. However, such quantities are usually calculated as genome-wide summaries and do not fully describe the spatial distribution of heterozygous and homozygous ancestry states along chromosomes.

This distinction is central for composite-breed management. Two animals with the same global ancestry proportion may not have the same genomic architecture. For a nominal Brangus animal, a Brahman ancestry proportion of 0.375 can be achieved through different combinations of Angus homozygous, heterozygous and Brahman homozygous ancestry states. These architectures may differ in ancestry heterozygosity, in the amount of Brahman homozygosity, and in the size and distribution of ancestry blocks along chromosomes. Consequently, animals that are equivalent in global breed composition may differ in their segregation behaviour when used as parents. From a breeding-management perspective, the relevant question is therefore not only whether a candidate sire or dam is nominal, but also what distribution of offspring ancestry is expected from a particular sire–dam pair.

This shift from individual selection to mating-specific evaluation is consistent with broader developments in animal breeding. Mating decisions can be formulated as optimization problems because large numbers of possible sire–dam combinations may differ in expected offspring performance, inbreeding, heterosis-related criteria or other breeding objectives [20]. In composite breeds, such approaches may also be relevant for managing breed composition.

In this study, we used controlled simulations to examine how alternative genomic architectures can affect the risk of producing non-nominal offspring in a simulated composite cattle breed. We modelled Brangus formation under two alternative pathways that lead to the same expected Brahman ancestry proportion of 0.375 but generate different ancestry-state configurations. We first evaluated how these pathways affect individual ancestry composition, ancestry heterozygosity and the genomic architecture of nominal catalogue sires. We then examined family-level segregation by simulating offspring from specific sire–dam pairs and decomposing non-nominal risk into two components: the distance between the expected family mean and the target ancestry, and the block-weighted segregation structure of the parental genomes. Finally, we evaluated whether mating-specific genomic information could improve sire allocation under alternative catalogue ancestry intervals.

The objective of this study was not to reproduce the full complexity of real Brangus populations, but to develop a controlled framework for separating global ancestry proportion from segregating genomic architecture. We propose that, in composite-breed management, nominal ancestry should be interpreted not only as an individual attribute, but also in terms of the offspring distribution generated by specific matings. In this framework, the sire–dam pair becomes a key unit of control for managing breed composition and reducing the risk of producing non-nominal offspring.

## Methods

### Fixed breed founders

Simulations were performed using the R package AlphaSimR [21]. The simulation started from two founder populations representing Angus and Brahman ancestry. Each founder population consisted of 100 diploid individuals with 29 chromosomes, 100 biallelic loci per chromosome, and a genetic length of 1 Morgan per chromosome, giving a total of 2900 loci.

To create a fully controlled ancestry system, the two founder breeds were made completely differentiated at all loci. Angus genotypes were fixed as *g* = 0 (0/0) across the genome, whereas Brahman genotypes were fixed as *g* = 2 (1/1). Thus, the simulation began with two alternative fixed ancestry states, and heterozygous loci generated in subsequent crosses were coded as *g* = 1 (0/1).

This fixed-founder design was intentionally idealized. Real Angus and Brahman populations are not fully fixed for alternative alleles, and real breed ancestry would need to be estimated from genotype data. However, the purpose of the simulation was not to reproduce the full complexity of real Angus and Brahman populations, but to isolate the effects of genomic architecture, recombination and segregation under controlled conditions. Under this design, the simulated Brahman ancestry proportion directly reflects the proportion of Brahman alleles across the genome, and all variation among descendants arises from recombination, Mendelian segregation and parental genomic structure, rather than from incomplete differentiation between founder breeds.

### Crossing schemes

A nominal Brangus individual is expected to carry 5/8 Angus and 3/8 Brahman ancestry. We simulated two alternative pathways leading to the same expected Brahman ancestry proportion of 0.375 (Fig. 1). These pathways are referred to throughout the manuscript as the direct pathway (D) and the indirect pathway (I).

**Fig. 1:**
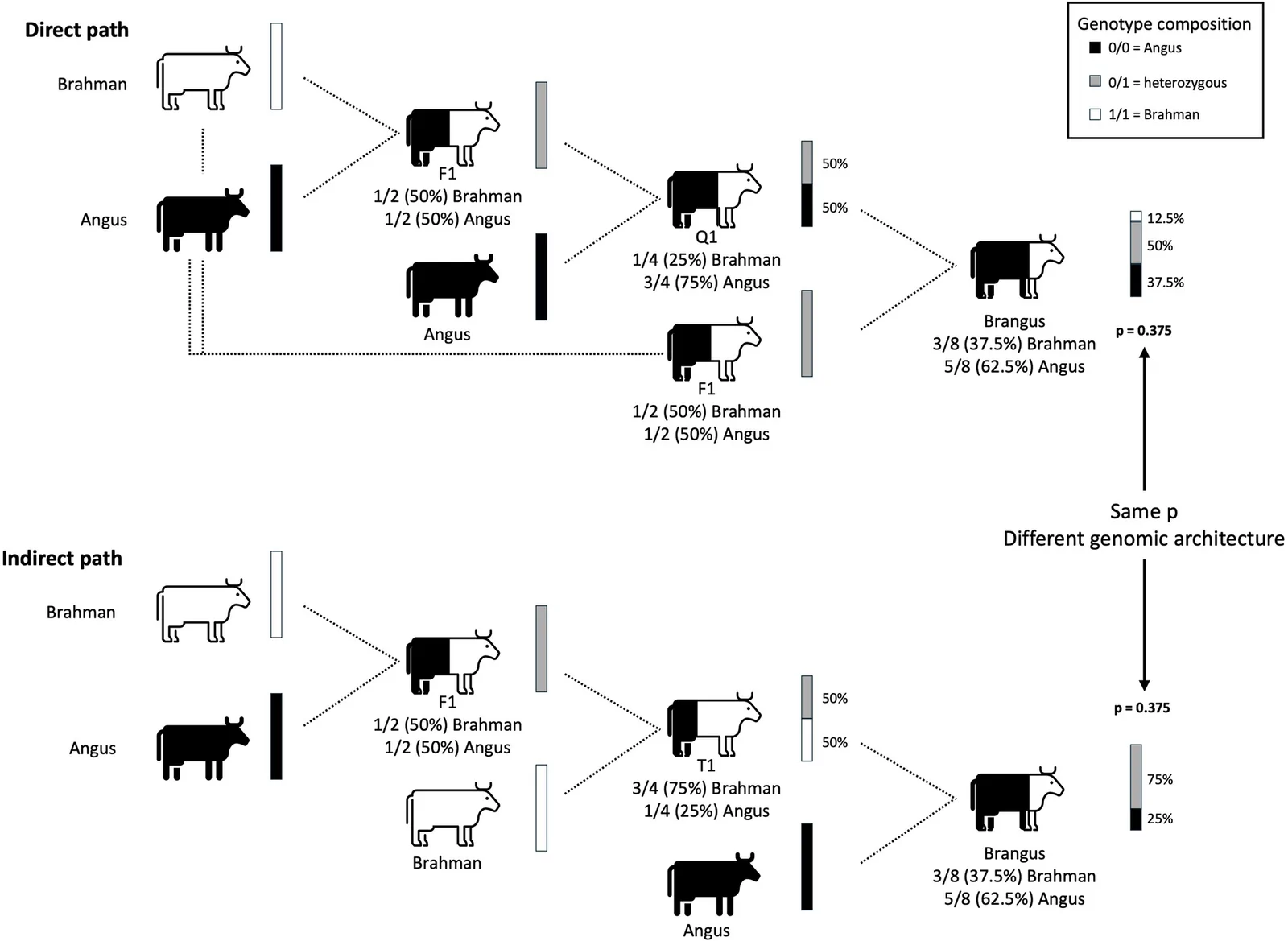
Alternative pathways to Brangus formation. The direct (top) and indirect (bottom) pathways start from Angus (0/0) and Brahman (1/1) founders and have the same expected final Brahman ancestry proportion of 0.375. F1 denotes the first filial generation; Q1 and T1 denote offspring from F1 × Angus and F1 × Brahman crosses, respectively. Stacked bars show the proportions of Angus homozygous (0/0), heterozygous (0/1) and Brahman homozygous (1/1) loci

In the direct pathway, Angus and Brahman founders were first crossed to generate individuals of the first filial generation (F1). Because the founder breeds were fixed for alternative alleles, all F1 individuals were heterozygous at all loci (*g* = 1). F1 individuals were then crossed with Angus founders to generate Q1 individuals, with an expected Brahman ancestry proportion of 0.25 and possible genotypes *g* ∈ {0,1}. Final Brangus individuals were generated by crossing Q1 individuals with independently generated Angus × Brahman F1 individuals. This pathway produces individuals with an expected average Brahman ancestry proportion of 3/8.

In the indirect pathway, Angus and Brahman founders were first crossed to generate F1 individuals, as above. F1 individuals were then crossed with Brahman founders to generate T1 individuals, with an expected Brahman ancestry proportion of 0.75 and possible genotypes *g* ∈ {1,2}. Final Brangus individuals were generated by crossing T1 individuals with Angus founders. This pathway also has an expected Brahman ancestry proportion of 3/8.

Thus, both pathways converge to the same expected global ancestry proportion, but they generate different genotype spaces. In the direct pathway, final individuals can contain Angus homozygous, heterozygous and Brahman homozygous loci (*g* ∈ {0,1,2}). In contrast, in the indirect pathway, final individuals can contain only Angus homozygous and heterozygous loci (*g* ∈ {0,1}), because the final cross is made between T1 and Angus individuals. These differences in accessible genotype states provide the basis for the pathway-dependent genomic architectures analysed in the subsequent sections.

### Genome composition and ancestry metrics

Genotypes were coded as the number of Brahman alleles carried by individual *i* at locus *j*: $\:{g}_{ij}\in\:\left\{\mathrm{0,1},2\right\},$

where *g*_*ij*_ = 0 denotes Angus homozygosity, *g*_*ij*_ = 1 denotes heterozygosity, and *g*_*ij*_ = 2 denotes Brahman homozygosity. The Brahman ancestry proportion of individual *i* was defined as:

1 $$p_i = \frac{1}{L} \sum_{j=1}^{L} \frac{g_{ij}}{2}$$

where *L* is the total number of loci. The target Brangus composition was defined as p^*^= 0.375 corresponding to the nominal 3/8 Brahman ancestry proportion. The absolute deviation of individual *i* from this target was calculated as *d*_*i*_ = ∣*p*_*i*_ − p^*^∣.

For a given tolerance threshold *δ*, the proportion of individuals exceeding that deviation from the target was calculated as:

2 $$\:{P}_{non}=P\left({d}_{i}>\delta\:\right)=\frac{1}{N}\sum\:_{i=1}^{N}1\left(\left|{p}_{i}-{p}^{*}\right|>\delta\:\right).\:$$

where *N* is the number of individuals and ***1(.)*** is the indicator function. Unless otherwise specified, the nominal Brangus interval was defined as 0.350 ≤ *p*_*i*_ ≤ 0.400, corresponding to p^*^ ± 0.025.

To characterize genomic architecture beyond global ancestry proportion, each genome was decomposed into three ancestry-state components: the proportion of Angus homozygous loci (*A*_*i*_), heterozygous loci (*H*_*i*_), and Brahman homozygous loci (*B*_*i*_):

3 $$A_i = \frac{1}{L}\sum_{j=1}^{L}\mathbb{1}(g_{ij}=0),$$

4 $$H_i = \frac{1}{L}\sum_{j=1}^{L}\mathbb{1}(g_{ij}=1),$$

and

5 $$B_i = \frac{1}{L}\sum_{j=1}^{L}\mathbb{1}(g_{ij}=2).$$

These components satisfy: *A*_*i*_ + *H*_*i*_ + *B*_*i*_ = 1 and the Brahman ancestry proportion can be written as *p*_*i*_ = *B*_*i*_ + ½ *H*_*i*_.

This decomposition separates global ancestry proportion from genomic architecture, allowing individuals with the same *p*_*i*_ to differ in the relative contribution of heterozygous and homozygous ancestry states.

Because founder breeds were fixed for alternative ancestry states in the simulation, the component *H*_*i*_ directly represents ancestry heterozygosity. We therefore use *H*_*i*_ for the formal ancestry-state decomposition and refer to the same quantity as ancestry heterozygosity when interpreting the results.

### Generation of catalogue sires and commercial female populations

To reflect a simplified commercial breeding setting, sire catalogues and commercial female populations were generated differently. Catalogue sires were assumed to represent selected Brangus breeding animals and were therefore required to fall within the nominal ancestry interval. For each pathway, 5000 final candidate males were simulated. Candidate sires were filtered to retain only individuals with Brahman ancestry proportion within 0.350 ≤ *p*_*i*_ ≤ 0.400. From the eligible individuals, 100 sires per pathway were randomly selected to form the direct-derived and indirect-derived sire catalogues.

In contrast, commercial female populations were not filtered by ancestry proportion. For each pathway, 1000 females were sampled directly from the corresponding simulated final population, retaining the full range of genomic outcomes generated by that breeding route. This asymmetry was intended to mimic a practical breeding scenario in which catalogue sires are more strongly selected and standardized, whereas commercial female populations may retain broader variation due to breeding history, recombination and segregation.

The resulting design generated two types of populations for subsequent analyses: filtered sire catalogues, standardized with respect to global Brahman ancestry, and unfiltered commercial female populations, which retained pathway-dependent variation in both ancestry proportion and genomic architecture.

### Mixed inter se mating and population-size sensitivity analysis

To evaluate whether pathway-dependent ancestry architectures persisted after direct- and indirect-derived animals were combined, we performed an additional mixed *inter se* mating analysis. A nominal foundation population was generated by sampling equal numbers of direct- and indirect-derived animals within the nominal ancestry interval 0.350 ≤ *p*_*i*_ ≤ 0.400, with 50% of each sex derived from the direct pathway and 50% from the indirect pathway.

To assess whether the mixed *inter se* dynamics were primarily driven by finite-population drift, the analysis was extended to 100 generations and repeated across three population-size scenarios. The Small scenario consisted of 200 females and 50 sires, the Medium scenario consisted of 1000 females and 250 sires, and the Large scenario consisted of 1500 females and 375 sires. In all scenarios, the mixed population was maintained under random *inter se* mating with overlapping generations and 25% replacement of the female and sire pools per generation.

Parents were sampled from the current adult population before removal. Dams were sampled without replacement, whereas sires were sampled with replacement, allowing repeated sire use. New offspring replaced the selected outgoing females and sires and became eligible as parents in the following generation.

For each generation, we recorded mean Brahman ancestry, the standard deviation (SD) of Brahman ancestry proportion, the proportion of animals outside the nominal interval, and the mean ancestry-state components *A*, *H* and *B*. We also recorded the realised number of contributing sires in each generation. As a descriptive population-size reference, an approximate realised effective size was calculated as 4*N*_*m*_*N*_*f*_/(*N*_*m*_+*N*_*f*_), where *N*_*m*_ was the realised number of contributing sires and *N*_*f*_ was the number of females in the scenario. This value was used only to compare the population-size scenarios and was not treated as a full estimate of effective population size under the overlapping-generation design.

Because displacement of the replicate mean contributes to the observed non-nominal frequency, we also calculated a centred non-nominal frequency to isolate the component attributable to within-population ancestry dispersion. For each replicate and generation, individual ancestry values were recentred to the target ancestry proportion as *p*_*centred, i*_ = *p*_*i*_ − $\:{\stackrel{-}{p}}_{rep}$ + 0.375, where $\:{\stackrel{-}{p}}_{rep}$ is the mean Brahman ancestry proportion of that replicate-generation. The corresponding centred deviation from the target was defined as *d*_*centred, i*_ = ∣*p*_*centred, i*_ − p^*^∣. The centred non-nominal frequency was then calculated as

6 $$P_{\mathrm{non\mathrm{-}centred}}= P\left(d_{\mathrm{centred},i} > \delta\right)= \frac{1}{N}\sum_{i=1}^{N}\mathbb{1}\left(\left|p_{\mathrm{centred},i}-p^*\right| > \delta\right).$$

Each population-size scenario was simulated in five independent replicates, and generation-specific summaries were computed as averages across replicates. Hardy-Weinberg reference values for q = 0.375 were used only as reference values for ancestry-state frequencies, namely *H* = 2*q*(1 − *q*) for heterozygous ancestry states and *B* = *q*^*2*^ for Brahman-homozygous ancestry states.

### Family-level simulations of nominal-parent crosses

To evaluate whether parental genomic architecture is associated with the distribution of offspring ancestry within a specific mating, we moved from individual-level and herd-level simulations to a controlled family-level design. In this analysis, a family was defined as the set of daughters (offspring) generated from a single sire–dam pair. Both sires and dams were required to be nominal Brangus animals, defined as individuals with Brahman ancestry proportion within the interval 0.350 ≤ *p*_*i*_ ≤ 0.400. This restriction was used to remove the confounding effect of non-nominal parents and to test whether nominal parents can nevertheless differ in the probability of producing non-nominal offspring.

Four mating scenarios were generated according to the formation pathway of the dam and sire, respectively: DD, DI, ID and II. The first letter denotes the pathway of the dam and the second letter denotes the pathway of the sire. Thus, DD represents direct-derived dams mated to direct-derived sires, DI represents direct-derived dams mated to indirect-derived sires, ID represents indirect-derived dams mated to direct-derived sires, and II represents indirect-derived dams mated to indirect-derived sires.

For each scenario, 1000 independent sire–dam pairs were sampled, and 100 daughters were simulated per pair. Thus, each family generated an empirical distribution of 100 offspring, from which family-level mean ancestry, variance and non-nominal risk were estimated.

For each offspring *o* in family *f*, the Brahman ancestry proportion was calculated as *p*_*o, f*_ using Eq. (1). For family *f*, the observed mean Brahman ancestry was:

7 $$\overline{p}_f = \frac{1}{n_f} \sum_{o=1}^{n_f} p_{o,f}$$

and the observed within-family variance was:

8 $$\:{s}_{p,f}^{2}=Var\left({p}_{o,f}\right)=\frac{1}{{n}_{f}-1}\sum\limits^{{n}_{f}}_{o=1}{\left({p}_{o,f}-{\overline{p}}_{f}\right)}^{2}$$

where *n*_*f*_ = 100 is the number of offspring simulated for family *f*.

The proportion of non-nominal daughters in family *f* was defined as:

9 $$\:{P}_{non,f}=\frac{1}{{n}_{f}}\sum\limits^{{{n}_{f}}}_{o=1}1\left({p}_{o,f}<0.350\cup\:{p}_{o,f}>0.400\right).\:\:$$

### Expected offspring ancestry from parental genotypes

For each sire–dam pair, we calculated the expected offspring ancestry composition directly from parental genotypes. This expectation represents the deterministic component of the cross, i.e. the expected mean offspring composition before Mendelian sampling.

At each locus, the dam and sire genotypes can form nine joint parental states:

0 × 0, 0 × 1, 0 × 2, 1 × 0, 1 × 1, 1 × 2, 2 × 0, 2 × 1, 2 × 2.

For each family, we computed the genome-wide frequency of each joint parental state. Let *P*_*ab, f*_ denote the proportion of loci in family *f* (i.e., a sire-dam pair) for which the dam has genotype a and the sire has genotype b, with a, b ∈ {0,1,2}.

The expected proportion of Angus homozygous loci among offspring from family *f* was:

10 $$\:{E[A}_{f}]={P}_{00,f}+\frac{1}{2}\left({P}_{01,f}+{P}_{10,f}\right)+{\frac{1}{4}P}_{11,f}.\:\:\:$$

The expected proportion of heterozygous loci was:

11 $$\begin{aligned}E[H_f]&= \frac{1}{2}\left(P_{01,f}+P_{10,f}\right)+ \left(P_{02,f}+P_{20,f}\right)+ \frac{1}{2}P_{11,f} \\&\quad + \frac{1}{2}\left(P_{12,f}+P_{21,f}\right) \\&= \frac{1}{2}\left(P_{01,f}+P_{10,f}+P_{11,f}+P_{12,f}+P_{21,f}\right) \\&\quad + P_{02,f}+P_{20,f}.\end{aligned}$$

The expected proportion of Brahman homozygous loci was:

12 $$\:{E[B}_{f}]={P}_{22,f}+\frac{1}{2}\left({P}_{12,f}+{P}_{21,f}\right)+{\frac{1}{4}P}_{11,f},\:$$

The expected mean Brahman ancestry of offspring from family *f* was then defined as:

13 $$\:{{\mu\:}_{arch,f}=E\left[{p}_{o,f}|f\right]=E\left[{\stackrel{-}{p}}_{f}\right]=E[B}_{f}]+\frac{1}{2}{E[H}_{f}],\:$$

where $\:{\mu\:}_{arch,f}$ is the expected family mean derived from parental genomic architecture.

Equivalently, for each dam–sire pair, the expected family mean can be calculated directly as $\:{\mu\:}_{arch,f}=\frac{1}{L}\sum\:_{l=1}^{L}\frac{{g}_{l}^{dam}+{g}_{l}^{sire}}{4}$.

### Parental joint-state blocks and block-weighted segregation potential

To derive the block-level variance weights, we first considered the expected Mendelian segregation variance at a single locus. At the single-locus level, the Brahman ancestry value of offspring *o* at locus *l* can be written as *p*_*o,l*_ = *g*_*o,l*_/2. This local value can take values 0, 0.5 or 1, corresponding to Angus homozygosity, heterozygosity and Brahman homozygosity, respectively.

For deterministic parental states (0 × 0, 0 × 2, 2 × 0, 2 × 2), the offspring genotype at that locus *l* is fixed and therefore, these states generate no segregation variance *v*_*l*_ = 0.

For parental states in which one parent is heterozygous and the other is homozygous at locus *l* (0 × 1, 1 × 0, 1 × 2, 2 × 1), the offspring Brahman ancestry value at that locus can take two possible values with equal probability. For example, in a 0 × 1 state, the Brahman ancestry value of offspring *o* is *p*_*o,l*_ = 0 or *p*_*o,l*_ = 0.5, each with probability 1/2, giving *E[p*_*o,l*_*]* = 0.25 and variance *v*_*l*_ = ½ x (0–0.25)^2^ + ½ x (0.5–0.25)^2^ = 1/16.

Finally, for heterozygote × heterozygote states (1 × 1), the Brahman ancestry value of offspring *o* can be *p*_*o,l*_ = 0, *p*_*o,l*_ = 0.50, or *p*_*o,l*_ = 1, each with probability ¼, ½ and ¼, respectively, giving *E[p*_*o,l*_*]* = 0.5 and variance *v*_*l*_ = 0.25 × (0–0.5)^2^ + 0.50 × (0.5–0.5)^2^ + 0.25 × (1–0.5)^2^ = 1/8.

The *v*_*l*_ values therefore correspond to the variance in Brahman ancestry contribution of locus *l* conditional on the parental genotype states, and can take the following conditional values:

14 $$\:{v}_{l}=\left\{\begin{array}{c}0\:\:for\:parental\:states\:\left\{0\times\:\mathrm{0,0}\times\:\mathrm{2,2}\times\:\mathrm{0,2}\times\:2\right\}\:\\\:\frac{1}{16}for\:parental\:states\:\left\{0\times\:\mathrm{1,1}\times\:\mathrm{0,1}\times\:\mathrm{2,2}\times\:1\right\}\\\:\frac{1}{8}\:for\:parental\:states\:\left\{1\times\:1\right\}\:\:\:\:\:\:\:\:\:\:\:\:\:\:\:\:\:\:\:\:\:\:\:\:\:\:\:\:\:\:\:\:\:\:\:\end{array}\right.\:\:$$

Since loci are not independently inherited, we considered the spatial structure of parental genotype states along chromosomes to predict within-family segregation variance. Consecutive loci with the same joint parental genotype state were grouped into parental joint-state blocks. Blocks were defined separately within each chromosome, so that no block could extend across chromosome boundaries.

For each sire–dam pair *f*, the parental genomes were decomposed into *K*_*f*_ parental joint-state blocks. These blocks were defined from the parental genotypes before simulating offspring. For each block *k*, we recorded the chromosome, start and end positions, block length, dam and sire genotype states, joint parental state, and the Mendelian variance associated with that state, *v*_*fk*_, as specified in Eq. (14). The relative genomic weight of block *k* in sire–dam pair *f* was defined as $\:{w}_{fk}=\frac{{L}_{fk}}{L},$ where *L*_*fk*_ is the number of loci in block *k* of sire-dam pair *f*, and *L* is the total number of loci. Note that if each block contained a single locus, the block-level variance assignment would reduce to the single-locus variance assignment.

Because genome-wide offspring ancestry can be viewed as a weighted average of block-level ancestry contributions, the block weights enter the variance approximation as squared weights. Thus, when each block was treated as a weighted unit contributing to offspring ancestry, the contribution of block *k* to the block-weighted segregation potential index was defined as $\:{w}_{fk}^{2}{v}_{fk}$, and the predictor for sire–dam pair *f* was:

15 $$\:{V}_{f}=\sum\limits^{{K}_{f}}_{k=1}{w}_{fk}^{2}{v}_{fk}.\:$$

This quantity was used as a *block-weighted segregation potential index* rather than as a formal analytical estimator of within-family ancestry variance. It summarises the amount and spatial concentration of parental joint-state configurations capable of generating ancestry segregation, while recombination, covariance among linked segments and stochastic chromosome transmission were captured empirically through explicit offspring simulation. Nevertheless, it captures a key feature of the parental architecture: the contribution of variance-generating genomic regions depends not only on their total genomic proportion, but also on their distribution across blocks. This can be seen with the following example: because block contributions scale with squared genomic weight $\:{w}_{k}^{2}$, large variance-generating blocks contribute disproportionately more than fragmented blocks of equivalent total length. For instance, a single block covering 40% of the genome in a *v*_*fk*_ = 1/8 state contributes: 0.4^2^ × 1/8 = 0.020, whereas four independent blocks each covering 10% of the genome contribute: 4 × (0.1^2^ × 1/8) = 0.005. Thus, both the amount and the spatial organization of high-variance states influence the expected segregation potential of a mating.

For the statistical model (next section), the block-weighted segregation potential index was also decomposed into two components:

16 $$\:{V}_{f,1/16}=\sum\limits_{{k:v}_{k}=1/16}{w}_{fk}^{2}\frac{1}{16},\:\:$$

and

17 $$\:{V}_{f,1/8}=\sum\limits_{{k:v}_{k}=1/8}{w}_{fk}^{2}\frac{1}{8}.\:\:\:$$

These two indexes distinguish block-weighted segregation potential generated by single-heterozygote parental states from variance generated by heterozygote × heterozygote states.

In addition, we recorded the simple genomic proportions in each index class *Prop*_*f,0*_, *Prop*_*f,1/16*_, *Prop*_*f,1/8*_.

These variables represent the fraction of the genome lying in blocks with variance 0, 1/16, and 1/8, respectively. Unlike the block-weighted segregation potential indexes, these proportions do not account for squared block length and therefore do not capture the disproportionate effect of long segregating blocks.

Finally, for each family, we calculated the distance between the expected offspring mean and the target Brangus ancestry *d*_*f*_ = ∣*µ*_*arch, f*_ – p^*^∣.

This variable captures the centring component of risk: even if segregation variance is low, a family whose expected mean is shifted away from the target may still produce a high proportion of non-nominal daughters.

### Exploratory screening model for family-level non-nominal risk

To inform the subsequent heuristic sire-allocation procedure (next section), we first evaluated whether simple, readily computable descriptors of parental ancestry architecture were associated with family-level non-nominal offspring risk. We considered the distance of the expected family mean from the nominal target, *d*_*f*_, and the block-weighted segregation potential terms, *V*_*f,1/8*_ and *V*_*f,1/16*_ as candidate architecture-derived covariates. This analysis was intended as an exploratory screening step to identify variables for the heuristic search, not as a complete generative model of offspring segregation.

For each family *f*, the response was the number of non-nominal offspring *n*_*non, f*_ out of the *n*_*f*_ = 100 simulated offspring from the corresponding sire–dam pair. An offspring was classified as non-nominal when its Brahman ancestry proportion fell outside the interval 0.350 ≤ *p*_*o, f*_ ≤ 0.400.

The response was modelled as a binomial-type response with denominator *n*_*f*_, using a quasibinomial error structure to account for overdispersion. The explanatory variables were derived from the parental genotypes. The first predictor was the distance between the expected family mean and the target Brahman ancestry, *d*_*f*_, as described earlier. Families whose expected mean is farther from the target are expected to have a higher probability of producing daughters outside the nominal interval. The second and third predictors were the two block-weighted segregation potential indexes: *V*_*f,1/8*_ and *V*_*f,1/16*_ (Eqs. 17 and 16), which correspond to the contributions from parental joint-state blocks with Mendelian variance 1/8, and 1/16, respectively. These terms capture the segregation component of risk generated by the parental genomic architecture.

The family-level risk model was therefore specified as:

18 $$\:{logit(P}_{non,f})\sim\:{\beta\:}_{0}+{\beta\:}_{1}{d}_{f}+{\beta\:}_{2}{V}_{f,1/8}+{\beta\:}_{3}{V}_{f,1/16}.\:\:$$

The model was fitted separately within each mating scenario (DD, DI, ID and II), because the four scenarios differed systematically in their accessible parental architectures and in the distribution of the predictors. Scenario-specific models therefore allowed the association between risk and parental genomic structure to be evaluated within each pathway combination, rather than assuming a single common relationship across structurally different mating types.

We also evaluated, as a sensitivity analysis, an extended exploratory model including *d*_*f*_ × *V*_*f,1/8*_ and *d*_*f*_ × *V*_*f,1/16*_ interaction terms, because such interactions may be biologically plausible.

Preliminary binomial generalized linear models showed mild overdispersion. Therefore, inference was based on quasibinomial generalized linear models with a logit link. Overdispersion was assessed using the Pearson residual dispersion ratio:

19 $$\widehat{\phi}= \frac{1}{df_{\mathrm{res}}}\sum_f r_{\mathrm{Pearson},f}^{2}$$

where *df*_*res*_ is the residual degrees of freedom. Dispersion estimates are reported for each scenario-specific model.

Model coefficients are presented on the logit scale. Positive coefficients indicate an increase in the log-odds of producing a non-nominal daughter as the corresponding predictor increases. The distance term was interpreted as the effect of displacement of the expected family mean from the target, whereas *V*_*f,1/8*_ and *V*_*f,1/16*_ were interpreted as the effects of high- and intermediate-variance parental block structures, respectively.

### Sire allocation under alternative catalogue ancestry intervals

To evaluate whether mating-specific genomic information could improve nominal daughter yield, we performed a proof-of-concept sire-allocation analysis. This analysis was designed to represent a simplified commercial setting in which both dams and candidate sires may deviate from the target Brahman ancestry. The allocation criterion considered only mate-specific non-nominal offspring risk derived from ancestry architecture; genetic merit, inbreeding, reproductive constraints and economic traits were not included.

A fixed mixed commercial dam population was generated by sampling 1000 females from the pooled direct- and indirect-derived commercial female populations. These dams were not filtered by ancestry proportion, so that the population retained the full range of genomic outcomes generated by the two breeding pathways.

Candidate sires were sampled from the full pool of 5000 direct-derived and 5000 indirect-derived final candidate sires. Three sire catalogues were generated according to increasing ancestry tolerance around the target Brahman ancestry of 0.375. The strict catalogue included sires within 0.375 ± 0.025, the moderate catalogue included sires within 0.375 ± 0.05, and the broad catalogue included sires within 0.375 ± 0.100. For each catalogue, 100 sires were randomly sampled from the eligible candidate pool. These three catalogues were intended to represent increasing levels of deviation around the target ancestry.

For each sire catalogue, two allocation strategies were compared. In the random sire-allocation strategy, each of the 1000 dams was assigned one sire sampled at random from the catalogue, with replacement, and one daughter was generated per mating. Sires could therefore be used more than once.

In the heuristic-assisted simulation-informed strategy, sire choice was performed separately for each dam. First, all 100 candidate sires in the catalogue were ranked for that dam using a heuristic based on two quantities calculated from the parental genotypes: the absolute distance between the expected family mean Brahman ancestry and the target, *d*_*f*_, and the block-weighted segregation potential index, *V*_*f*_, both calculated as described earlier for the family-level analysis. Candidate matings, *m*, were ranked separately by *d*_*f*_ and *V*_*f*_, and a combined heuristic score was computed as the sum of the two ranks *H*_*m*_ = rank(*d*_*f*_) + rank(*V*_*f*_). Lower values of *H*_*m*_ indicated matings expected to be better centred on the target and to have lower segregation potential.

For each dam, the 10 sires with the lowest heuristic scores were retained as a shortlist. Each shortlisted mating was then evaluated by direct simulation of 50 probe offspring. The empirical non-nominal risk of each candidate mating was calculated as the proportion of probe offspring outside the nominal Brangus interval 0.350 ≤ *p*_*i*_ ≤ 0.400. The sire with the lowest empirical non-nominal risk was selected for that dam. In the case of ties, one of the tied sires was selected at random. After sire selection, one final independent offspring was generated from the selected mating and used for the comparison with random allocation of sires to dams.

For each catalogue and allocation strategy, we recorded the mean and standard deviation of offspring Brahman ancestry, the number and proportion of nominal and non-nominal offspring, the number of unique sires used, and the maximum number of offspring assigned to any single sire. Gains were expressed as the reduction in the number and proportion of non-nominal offspring achieved by the heuristic-assisted simulation-informed strategy relative to random allocation within the same sire catalogue.

All analyses were performed in R [22].

**Table 1:** Symbols and ancestry metrics

Symbol	Meaning
*p*_*i*_	realised Brahman ancestry proportion of individual *i*
*p* ^*^	target Brahman ancestry proportion: 0.375
$\:{\stackrel{-}{p}}_{rep}$	mean Brahman ancestry proportion of a replicate-generation
*p*_*centred,i*_	Brahman ancestry proportion of individual *i* after recentring the replicate-generation mean to p^*^
*d*_*i*_	absolute deviation of *p*_*i*_ from p^*^
*d*_*centred,i*_	absolute deviation of *p*_*centred,i*_ from p^*^
*P*_*non*_	proportion of individuals outside the nominal ancestry interval
*P*_*non,centred*_	proportion of individuals outside the nominal ancestry interval after recentring the replicate-generation mean to p^*^
*p*_*o,f*_	realised Brahman ancestry proportion of offspring *o* in family *f*
$\:{\stackrel{-}{p}}_{f}$	observed mean offspring Brahman ancestry in family *f*
$\:{s}_{p,f}^{2}$	observed within-family variance in offspring Brahman ancestry
*µ*_*arch,f*_	expected family mean from parental genotypes
*d*_*f*_	distance of *µ*_*arch,f*_ from target
*v*_*fk*_	expected Mendelian segregation variance assigned to block *k*
*w*_*fk*_	relative genomic weight of block *k*
*V*_*f*_	block-weighted segregation potential index
*V*_*f,1/16*_, *V*_*f,1/8*_	components of *V*_*f*_ by variance class
*P*_*non,f*_	proportion of non-nominal offspring in family *f*

## Results

The results are presented in a sequence of analyses designed to move from broad population-level patterns to mating-specific evaluation. First, we evaluated whether two alternative formation pathways leading to the same expected Brahman ancestry proportion generated equivalent individual ancestry distributions and ancestry heterozygosity. Second, we examined whether filtering animals into the nominal Brangus interval was sufficient to remove pathway-dependent differences in genomic architecture. Third, we used simulated commercial herd renewal to test whether the use of nominal catalogue sires was sufficient to maintain commercial female populations within the nominal ancestry interval over generations.

We then moved from population and herd-level analyses to a controlled family-level design. In this part, we asked whether nominal sire–dam pairs differed in their probability of producing non-nominal offspring, and whether that risk was associated with the expected family mean and the block-weighted segregation structure of the parental genomes. Finally, we used these mating-specific predictors in a proof-of-concept sire allocation analysis to test whether genomic information could improve nominal offspring yield under alternative sire catalogue ancestry intervals.

### Distribution of ancestry composition and heterozygosity under alternative breeding pathways

We first compared the individual-level ancestry composition and ancestry heterozygosity generated by the D and I formation pathways, both of which have the same expected Brahman ancestry proportion in the final generation (p^*^ = 0.375; Fig. 1).

At the population level, both pathways produced mean Brahman ancestry proportions close to the nominal target. Across 100 simulated final individuals per pathway, the mean ancestry proportion was $\:\stackrel{-}{p}$ = 0.378 for the D pathway and $\:\stackrel{-}{p}$ = 0.373 for the I pathway. Thus, both routes achieved the expected composition in terms of their population mean. However, this similarity in mean ancestry masked clear differences in the distribution of individual ancestry proportions (Fig. 2a). The D pathway generated a broader distribution than the I pathway, with a higher standard deviation of Brahman ancestry proportion (SD = 0.040 versus 0.026). As a consequence, a larger fraction of D individuals departed from the nominal target. The proportion of individuals exceeding a deviation of ± 0.025 from p^*^ was 54% in the D pathway, compared with 32% in the I pathway. The same pattern was observed across additional tolerance thresholds, with the D pathway consistently showing a higher probability of deviation from the target composition [Additional file 1, Figure S1].

**Fig. 2:**
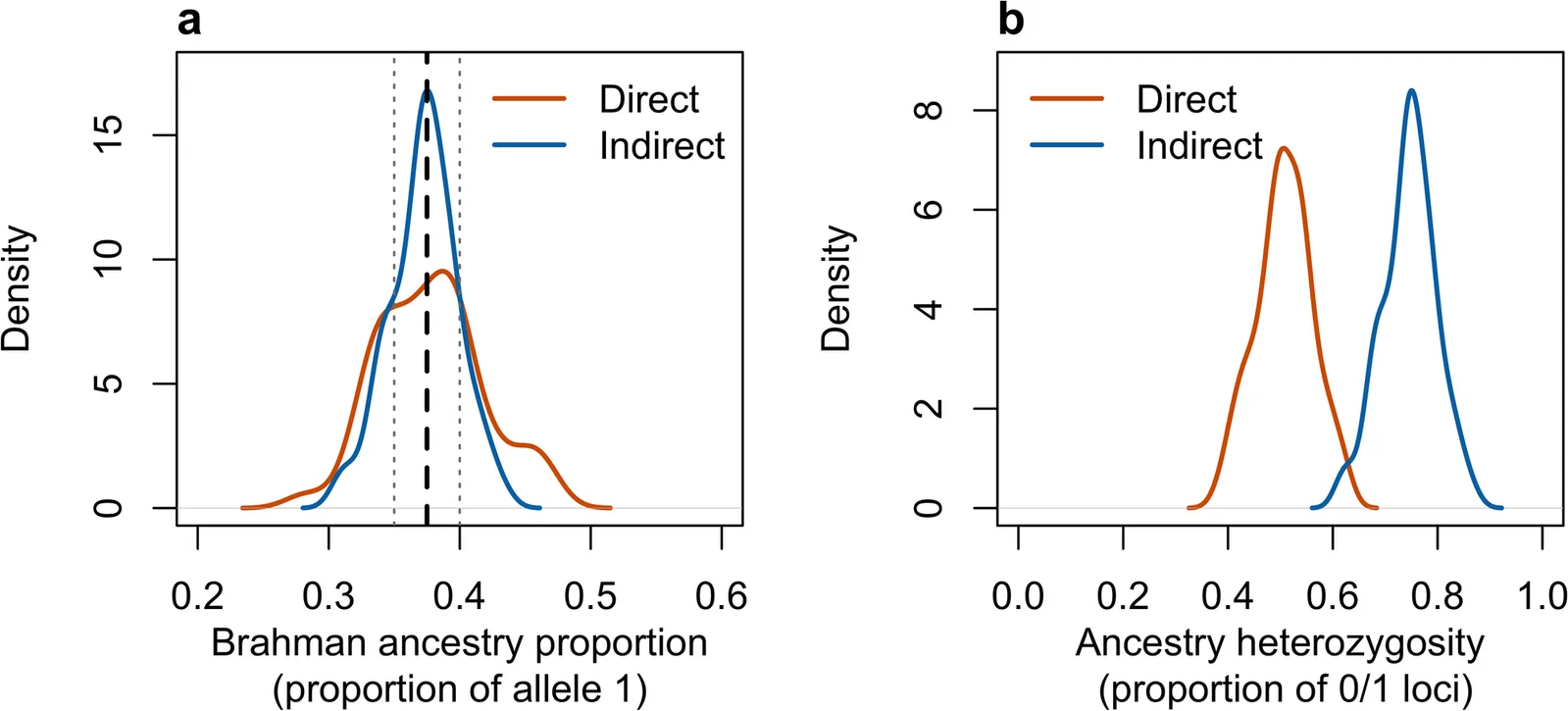
Distribution of Brahman ancestry proportion and ancestry heterozygosity under alternative breeding pathways. Distributions are shown for 100 individuals per pathway: (**a**) individual Brahman ancestry proportion and (**b**) ancestry heterozygosity. In panel a, the vertical dashed line marks the target ancestry proportion of 0.375, and the grey dashed lines delimit the nominal interval of 0.350–0.400

To determine whether these distributional differences were associated with differences in genomic architecture, we next examined ancestry heterozygosity, defined as the proportion of loci 0/1. The I pathway resulted in substantially higher heterozygosity ($\:\stackrel{-}{H}$ = 0.746) than the D pathway ($\:\stackrel{-}{H}$ = 0.507), reflecting distinct patterns of ancestry mixing across the genome (Fig. 2b). These values were consistent with the expected genotype structure generated by each pathway: the indirect route tends to produce genomes with high heterozygosity and little or no Brahman homozygosity, whereas the direct route allows a broader range of heterozygous and homozygous ancestry configurations.

Importantly, the overlap between the D and I heterozygosity distributions was restricted to a narrow interval, *H* = 0.614–0.628. This suggests that, once an animal has been confirmed as nominal based on *p*, its heterozygosity can provide additional practical information about the pathway by which that nominal composition was generated. For example, under the simulated framework, a nominal animal with *H* above the overlapping interval would be more consistent with an indirect origin, whereas a nominal animal with *H* below the overlapping interval would be more consistent with a direct origin. Thus, once genotype-derived ancestry estimates are available, the joint use of *p*_*i*_ and *H*_*i*_ may provide a useful preliminary genomic diagnostic for geneticists and breeding organisations when evaluating animals for purchase, selecting candidate sires, or assessing whether an animal’s genomic architecture is consistent with its expected formation pathway.

### Structural constraints and empirical genomic architectures of nominal Brangus

The differences observed at the population level (Fig. 2) have a direct consequence for breeding practice. In commercial settings, animals are generally expected to fall within a narrow range around the target breed composition. Thus, breeding programs effectively apply, or should apply, a selection filter on ancestry proportion before animals are used as breeding sires.

To mimic this process, we constructed pathway-specific catalogues of sires by sampling 100 individuals within the nominal interval of Brahman ancestry, 0.350 ≤ *p*_*i*_ ≤ 0.400, out of 5000 simulated candidate sires. This filtering step transformed the broad pathway-dependent distributions observed in Fig. 2 into two sets of sires that were comparable in terms of global Brahman ancestry proportion.

However, selecting on *p*_*i*_ alone does not uniquely define the genomic configuration of these individuals. To explore the range of genomic configurations for the nominal *p*_*i*_ interval, we decomposed each genome into the proportion of Angus homozygous loci (*A*), heterozygous loci (*H*), and Brahman homozygous loci (*B*) [Eqs. (3), (4) and (5)]. Given that Brahman ancestry proportion is defined as *p*_*i*_ = *B*_*i*_ + ½ *H*_*i*_, fixing the exact target p^*^ = 0.375 implies the linear relationship *B*_*i*_ = 0.375 – ½ *H*_*i*_.

This constraint emerges directly from the definition of ancestry composition. Therefore, the exact nominal target does not correspond to a single genomic architecture, but to a continuum of possible combinations of heterozygous and Brahman homozygous ancestry states (Fig. 3a). The nominal interval 0.350 ≤ *p*_*i*_ ≤ 0.400 can be interpreted as a narrow band around this central constraint.

**Fig. 3:**
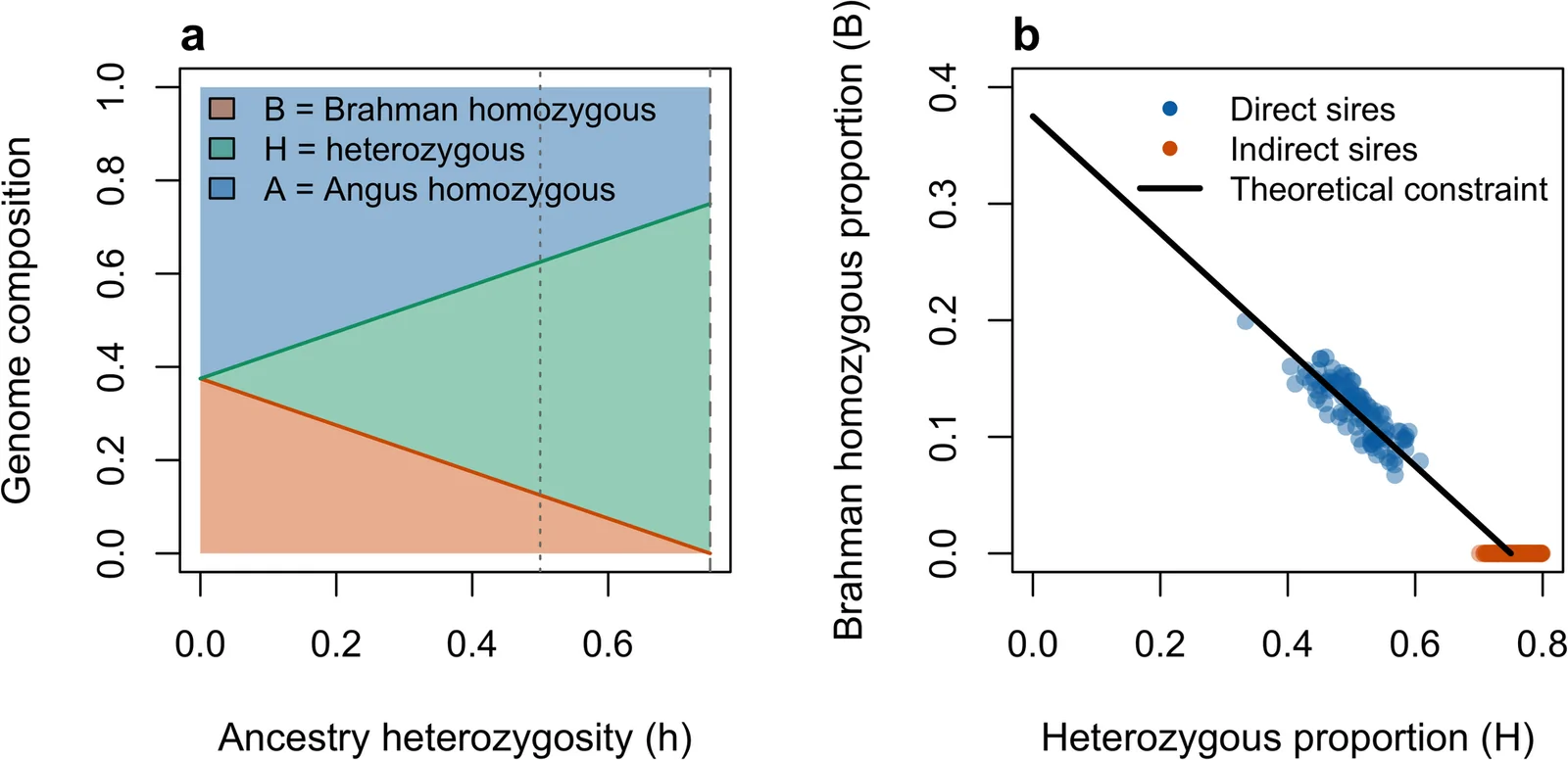
Structural constraint of nominal Brangus genomes and empirical architectures of catalogue sires. (**a**) Genomic compositions compatible with the target Brahman ancestry proportion of 0.375. A, H and B denote the proportions of Angus homozygous, heterozygous and Brahman homozygous loci, respectively, with B = 0.375 − 0.5H. Vertical dotted and dashed grey lines indicate the expected heterozygosity of direct-derived and indirect-derived sires, respectively. (**b**) Empirical architectures of 100 catalogue sires per pathway, selected within the nominal ancestry interval of 0.350 – 0.400

When the selected catalogue sires were projected onto this space of theoretical constraint, clear pathway-dependent differences remained evident (Fig. 3b). Sires derived from the direct pathway were distributed across a broader region of the constraint, with substantial variation in both heterozygosity and Brahman homozygosity. In contrast, sires derived from the indirect pathway were concentrated near the high-heterozygosity end of the space, with no Brahman homozygosity. This pattern reflects the restricted genotype space generated by the indirect crossing scheme, in which final individuals are largely composed of Angus homozygous and heterozygous loci.

Importantly, these differences arose despite both sets of sires satisfying the same nominal ancestry interval. In other words, filtering candidate sires on global Brahman ancestry standardized the catalogue with respect to *p*_*i*_, but it did not eliminate the structural differences imposed by the formation pathway. Nominal sires from the direct and indirect pathways can therefore remain genomically distinct even when they are indistinguishable by global ancestry proportion alone.

This distinction becomes particularly relevant when considering the female populations available for commercial mating. In contrast to the selected sire catalogues, the simulated commercial female populations (*n* = 1000) were not filtered by ancestry proportion and therefore retained the full variability generated by each pathway. Direct females spanned a wider range of Brahman ancestry values (*p*_*i*_ ∈ [0.217,0.521]), whereas indirect females showed a narrower distribution (*p*_*i*_ ∈ [0.292,0.449]), consistent with the pathway-level differences described above. Thus, the sire catalogue represents a filtered and standardized subset of each pathway, whereas the commercial female populations retain broader pathway-dependent variability.

Taken together, these results show that selection for nominal ancestry does not uniquely determine genomic architecture. Instead, breeding pathways define the region of the theoretical constraint space that is accessible, leading to structurally distinct sire catalogues even after strong filtering on global composition.

### Dynamics of nominal ancestry in simulated commercial herds

We next evaluated whether the use of nominal catalogue sires was sufficient to maintain commercial female populations within the nominal Brangus interval over successive generations. This analysis was designed to represent a practical herd-level scenario rather than a fully controlled family experiment. Starting from the unfiltered female populations described above, we divided each pathway-specific population (1000 females) into five commercial herds of 200 females. In each herd and generation, 25% of the females were replaced by 50 new daughters generated from random one-to-one matings between 50 unique commercial females and 50 unique nominal catalogue sires. This process was repeated for 10 generations.

Four renewal scenarios were considered: DD, DI, ID, and II, with the first letter indicating the female population pathway and the second letter indicating the sire catalogue pathway. Across all scenarios, mean Brahman ancestry remained close to the target value throughout the 10 generations [Additional file 2, Figure S2a]. Initial direct female herds had a mean ancestry of $\:\stackrel{-}{p}$ = 0.375, and after 10 generations the mean remained essentially unchanged in DD ($\:\stackrel{-}{p}$ = 0.375) while increasing slightly in DI ($\:\stackrel{-}{p}$ = 0.378). Initial indirect female herds also started close to the target ($\:\stackrel{-}{p}$ = 0.375), increasing modestly by generation 10 in both ID ($\:\stackrel{-}{p}$ = 0.380) and II ($\:\stackrel{-}{p}$ = 0.379). Thus, at the level of the population mean, all scenarios appeared to maintain the nominal Brangus composition after 10 generations.

However, this apparent stability in mean ancestry masked substantial differences at the individual level. At generation 0, the proportion of non-nominal females was higher in direct herds than in indirect herds, reflecting the broader ancestry distribution generated by the direct pathway. Direct herds started with 52.8% of females outside the nominal interval, whereas indirect herds started with 35.3% outside the interval. After 10 generations, the proportion of non-nominal females remained high in all scenarios [Additional file 2, Figure S2b]. It decreased to 43.4% in DD and to 49.6% in DI, but increased to 45.9% in ID and to 47.3% in II.

The direction of change depended on both the starting female population and the sire catalogue used. In direct herds, renewal with direct catalogue sires reduced the proportion of non-nominal females by 9.4% points, whereas renewal with indirect catalogue sires produced only a small reduction of 3.2% points. In contrast, indirect herds showed an increase in non-nominal females over generations, rising by 10.6% points in ID and by 12.0% points in II. These changes occurred despite the fact that all sire catalogues were composed exclusively of nominal sires.

Ancestry heterozygosity also changed markedly during the renewal process [Additional file 2, Figure S2c]. Direct herds showed only a moderate decrease in mean heterozygosity, from 0.501 at generation 0 to 0.470 in DD and 0.473 in DI at generation 10. In contrast, indirect herds showed a strong decline, from 0.749 at generation 0 to 0.489 in ID and 0.486 in II. This indicates that the highly heterozygous architecture generated by the indirect formation pathway was rapidly diluted under repeated commercial renewal.

At the final generation, non-nominal females were distributed on both sides of the target interval [Additional file 2, Figure S2d]. The relative contribution of below- and above-nominal animals differed among scenarios, but all scenarios retained a substantial fraction of females outside the accepted range. Final non-nominal proportions ranged from 43.4% in DD to 49.6% in DI, indicating that the use of nominal catalogue sires alone did not ensure maintenance of commercial herds within the nominal Brangus interval.

Overall, this commercial herd simulation shows that population mean ancestry can remain close to the target while a large proportion of individual females falls outside the nominal range. Therefore, monitoring only the average breed composition of a herd may be insufficient. The results suggest that practical Brangus management requires attention to individual-level ancestry distributions and parental genomic architecture, not only to the nominal status of selected sires.

### Mixed inter se mating produces a slow decline in ancestry variance across population-size scenarios

The commercial herd analysis suggested that pathway-dependent ancestry heterozygosity differences were progressively attenuated during repeated renewal, particularly in scenarios involving indirect-derived females [Additional file 2, Figure S2c]. To evaluate this point more directly, we added a mixed *inter se* mating analysis in which nominal direct- and indirect-derived animals were combined into a single finite breeding population and maintained for 100 generations under random Brangus-target × Brangus-target mating.

Three population-size scenarios were analysed: Small, Medium and Large (Fig. 4). Approximate realised effective size, calculated from the realised number of contributing sires, increased from 121 in the Small scenario to 602 and 907 in the Medium and Large scenarios, respectively. Across scenarios, mean Brahman ancestry remained close to the target value of 0.375 throughout the simulation, although replicate-to-replicate displacement of the mean was greater in the Small scenario (Fig. 4a). At generation 100, the mean absolute displacement from the target was 0.0087 in the Small scenario, compared with 0.0025 and 0.0027 in the Medium and Large scenarios.

**Fig. 4:**
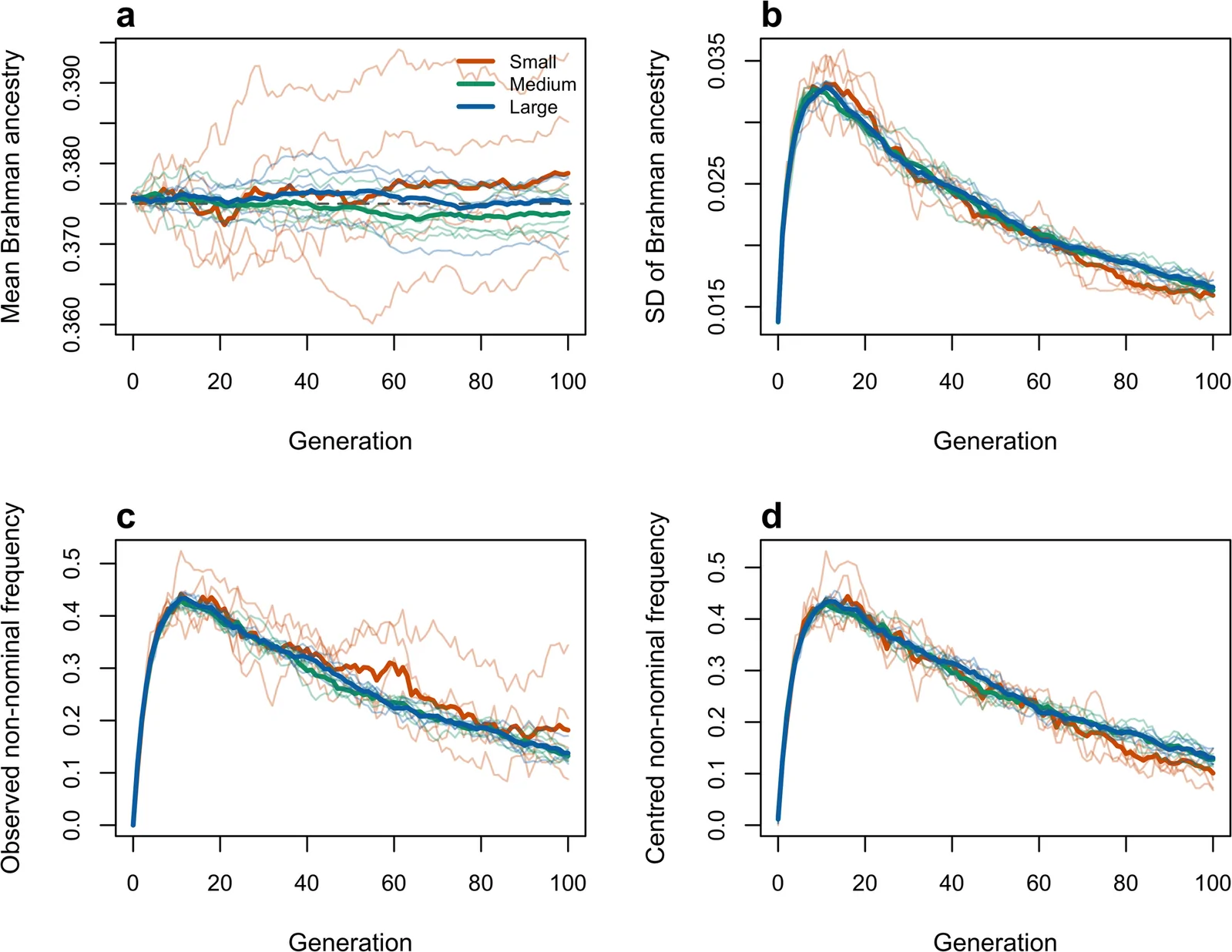
Mixed *inter se* mating analysis across population-size scenarios. Nominal animals from both formation pathways were combined and maintained for 100 generations under random inter se mating. Populations comprised 200 females and 50 sires (Small), 1000 females and 250 sires (Medium), or 1500 females and 375 sires (Large). Thin lines show five independent replicates; thick lines show their means. Panels show (**a**) mean Brahman ancestry proportion, (**b**) its standard deviation, (**c**) observed non-nominal frequency and (**d**) non-nominal frequency after recentring each replicate-generation to the target ancestry proportion of 0.375. The dashed horizontal line in panel a marks this target

In all scenarios, SD(*p*) increased rapidly during the first generations of mixed *inter se* mating, reached a maximum around generations 10–15, and then declined gradually up to generation 100 (Fig. 4b). Despite large differences in approximate realised effective size, SD(*p*) remained similar across scenarios at generation 100, reaching 0.0159, 0.0163 and 0.0166 in the Small, Medium and Large scenarios, respectively. For comparison, under an idealised independent-locus expectation with L = 2900 loci and target ancestry p^*^ = 0.375, the expected standard deviation is $\:\sqrt{{p}^{*}(1-{p}^{*})/2L}$ = 0.0064. Thus, the generation-100 values remained above the independent-locus reference.

Observed non-nominal frequency, *P*_*non*_ (Eq. (2)), also declined after its early maximum but remained non-negligible at generation 100: 0.182, 0.132 and 0.137 in the Small, Medium and Large scenarios, respectively (Fig. 4c). After recentring each replicate-generation to the target ancestry proportion, the corresponding centred non-nominal frequencies, *P*_*non, centred*_ (Eq. (6)), were 0.101, 0.127 and 0.131, respectively (Fig. 4d). Thus, replicate-mean displacement contributed to *P*_*non*_ in the Small scenario, whereas *P*_*non*_ and *P*_*non, centred*_ were similar in the Medium and Large scenarios. This indicates that non-nominal status in the larger scenarios was driven mainly by within-population ancestry dispersion, as reflected by SD(*p*), rather than by displacement of the replicate mean.

### Family-level segregation structure explains non-nominal offspring risk among nominal parents

The commercial herd simulations showed that using nominal catalogue sires was not sufficient to maintain all females within the nominal Brangus interval over successive generations. This raises a more fundamental question: if a sire is nominal, why can its use still produce a large proportion of non-nominal offspring? One possibility is that the nominal status of an individual parent is an incomplete descriptor of its reproductive behaviour. In other words, the relevant quantity may not be whether each parent falls within the nominal interval, but how the two parental genomes combine and segregate in a specific mating.

To address this question, we next moved from herd-level dynamics to a controlled family-level analysis. In this analysis, both sires and dams were required to be nominal (0.350 ≤ *p*_*i*_ ≤ 0.400), thereby removing the confounding effect of non-nominal commercial females. We then generated four nominal-parent mating scenarios according to the formation pathway of the dam and sire: DD, DI, ID, and II. For each scenario, 1000 independent sire–dam pairs were simulated, and 100 offspring were generated per pair. This design allowed us to ask whether matings between nominal parents differ in their probability of producing non-nominal offspring, and which properties of the parental genomes explain that risk.

Under this controlled design, all parental groups were closely centred around the target Brahman ancestry. Mean ancestry proportions were $\:\stackrel{-}{p}$ = 0.376 in direct sires, $\:\stackrel{-}{p}$ = 0.375 in direct dams, $\:\stackrel{-}{p}$ = 0.374 in indirect sires, and $\:\stackrel{-}{p}$ = 0.376 in indirect dams. Therefore, any differences in offspring risk among scenarios could not be attributed to systematic differences in the mean ancestry of the parental groups. Instead, they must arise from the way ancestry segregates from each specific sire–dam combination.

Despite this control, family-level outcomes differed markedly among scenarios. The mean proportion of non-nominal offspring followed a clear gradient (Eq. (9)): DD produced the lowest risk ($\:{\stackrel{-}{P}}_{non,f}$= 0.381), ID and DI were intermediate ($\:{\stackrel{-}{P}}_{non,f}$= 0.462 and 0.464), and II produced the highest risk ($\:{\stackrel{-}{P}}_{non,f}$ = 0.519). Thus, even when both parents were nominal and the parental groups were matched for mean ancestry, the pathway combination of the mating strongly affected the probability of producing non-nominal offspring (Fig. 5a).

**Fig. 5:**
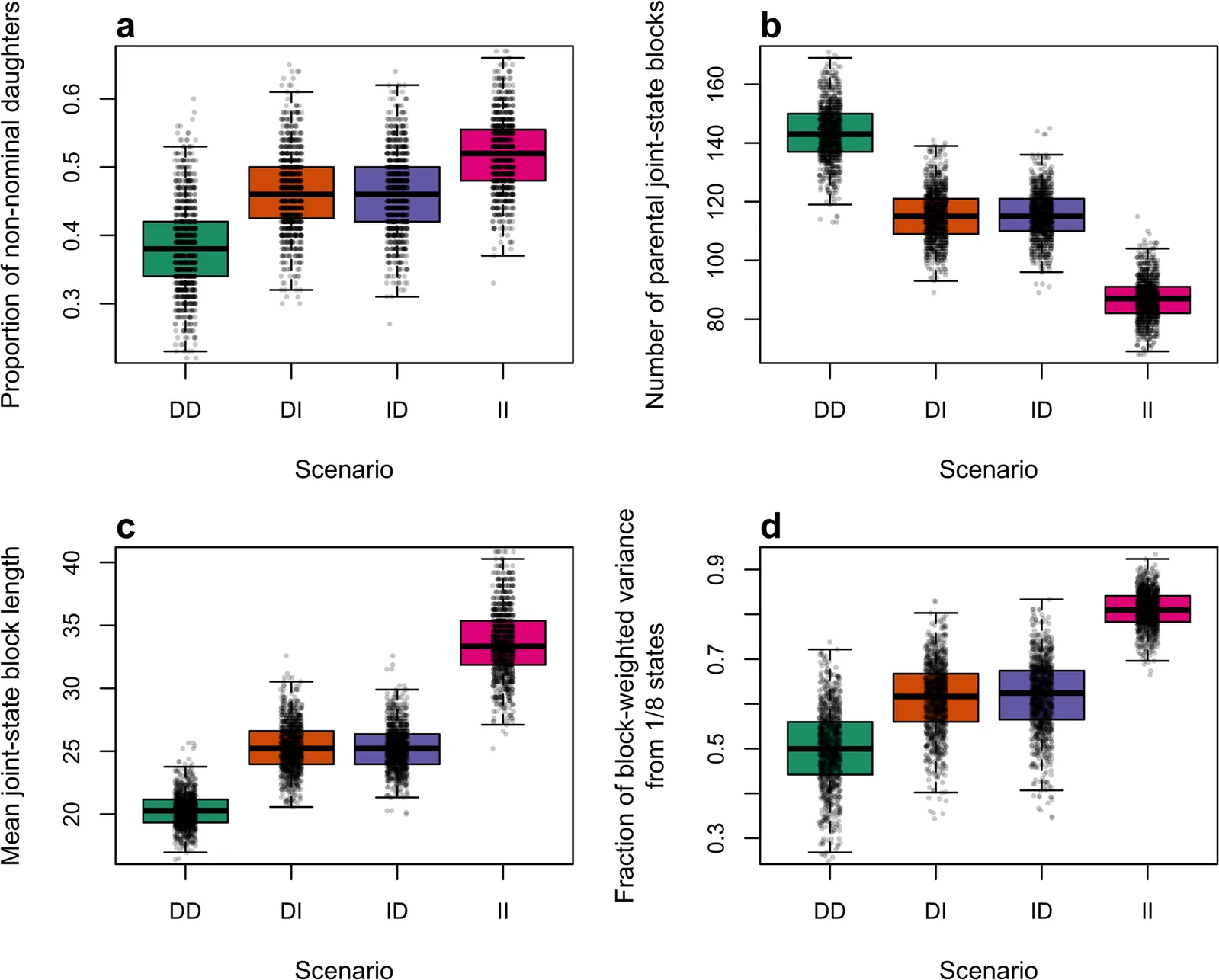
Family-level non-nominal risk and parental joint-state block structure among nominal-parent matings. Results are shown for 1000 independent sire–dam pairs per scenario, with 100 daughters simulated per pair. Scenario labels identify the dam pathway followed by the sire pathway: D, direct; I, indirect. Each point represents one sire–dam pair. Panels show (**a**) the proportion of daughters outside the nominal ancestry interval of 0.350–0.400, (**b**) the number of parental joint-state blocks, (**c**) mean block length and (**d**) the fraction of block-weighted segregation potential attributable to 1/8 states, in which both parents are heterozygous

These differences were not caused by shifts in the family mean. Observed family mean ancestry remained close to the target in all scenarios, with mean values, $\:{\stackrel{-}{p}}_{f}$, ranging from 0.375 to 0.376. The expected family mean calculated from parental genotypes, *µ*_*arch, f*_ (Eq. (13)), also closely matched the observed family mean, $\:{\stackrel{-}{p}}_{f}$ (Eq. (7)), with correlations between 0.938 and 0.967 and coefficients of determination (R²) between 0.881 and 0.936 [Additional file 3, Figure S3]. Therefore, the increased risk in II and the reduced risk in DD were not primarily due to directional displacement from the target, but to differences in dispersion around the expected family mean.

This raised the next question: if scenario differences were not mainly due to shifts in expected family mean, what feature of the parental genomes generated the observed differences in non-nominal risk? We hypothesized that the answer lies in the segregation structure of the parental genomes. Offspring do not inherit ancestry states as independent loci, but as chromosomal segments transmitted through meiosis. Therefore, the same global ancestry proportion can generate different offspring distributions depending on how variance-generating parental states are arranged along the genome.

To evaluate this, we decomposed each sire–dam pair into parental joint-state blocks, defined as consecutive genomic segments sharing the same parental genotype combination. For instance, a sequence of five consecutive loci with parental states 0 × 1, 0 × 1, 0 × 1, 1 × 1, 1 × 1 would be decomposed into two parental joint-state blocks: one defined by three consecutive 0 × 1 loci and one defined by two consecutive 1 × 1 loci, where *g* = 0 corresponds to Angus homozygosity (AA), *g* = 1 to ancestry heterozygosity (AB), and *g* = 2 to Brahman homozygosity (BB).

Different joint states have different expected contributions to offspring ancestry variance (Eq. (14)). Heterozygote × heterozygote blocks generate the highest segregation variance (*v*_*fk*_ = 1/8), single-heterozygote blocks generate intermediate variance (*v*_*fk*_ = 1/16), and fully homozygous combinations generate no segregation variance, (*v*_*fk*_ = 0). Because each block contributes to the offspring ancestry variance according to the square of its genomic weight ($\:{w}_{fk}^{2}$), larger high-variance blocks have a disproportionate effect on the expected segregation variance of the family. Details and examples are provided in the Methods section.

The four mating scenarios differed systematically in this block structure (Fig. 5b and c; Table 2). DD families showed the highest number of parental joint-state blocks and the shortest mean block length, indicating a more fragmented segregation structure. In contrast, II families showed fewer but longer blocks. The mixed scenarios, ID and DI, were intermediate and highly similar to each other. The contribution of high-variance 1/8 states (*V*_*f,1/8*_; Eq. (17)) also followed the same structural gradient: it was lowest in DD, intermediate in ID and DI, and highest in II (Fig. 5d; Table 2).

Having identified these structural differences, we then examined whether they matched the observed family-level variance. Mean variance in offspring Brahman ancestry (Eq. (8)) was lowest in DD (0.00072), intermediate in ID and DI (0.00106 and 0.00106), and highest in II (0.00142). The block-weighted segregation potential index *V*_*f*_ (Eq. (15)) followed the same ordering, increasing from 0.00078 in DD to approximately 0.00123 in ID and DI, and 0.00196 in II. Thus, the ranking of observed family variance and non-nominal risk mirrored the ranking of parental segregation structure (Table 2).

**Table 2:** Descriptive statistics of controlled nominal-parent mating scenarios^a^

Statistic	DD	DI	ID	II
Number of families	1000	1000	1000	1000
Daughters per family	100	100	100	100
Mean$\:{\stackrel{-}{p}}_{f}$	0.3755	0.3745	0.3760	0.3753
Mean *µ*_*arch,f*_	0.3756	0.3746	0.3761	0.3751
$\:{\stackrel{-}{d}}_{f}$	0.00810	0.00809	0.00805	0.00824
cor(*μ*_*arch, f*_ ~$\:{\stackrel{-}{p}}_{f}$)	0.967	0.953	0.945	0.938
*R*^*2*^ (*μ*_*arch,f*_ ~$\:{\stackrel{-}{p}}_{f}$)	0.936	0.908	0.894	0.881
Mean Var (*p*_*o,f*_)	0.00072	0.00106	0.00106	0.00142
Mean *V*_*f*_	0.00078	0.00123	0.00123	0.00196
Mean *V*_*f,1/8*_	0.00039	0.00076	0.00076	0.00159
Mean *V*_*f,1/16*_	0.00038	0.00047	0.00046	0.00037
Mean *V*_*f,1/8*_ / Mean *V*_*f*_	0.499	0.612	0.617	0.811
*R*^*2*^ (*V*_*f*_ ~ Var (*p*_*o,f*_))	0.257	0.096	0.089	0.037
Mean number of parental joint-state blocks	144	115	116	87
Mean parental joint-state block length (loci)	20	25	25	34
Mean number of 1/8 blocks	36	43	44	49
Mean number of 1/16 blocks	72	58	58	33
Mean number of 0-variance blocks	36	14	14	6
$\:{\stackrel{-}{P}}_{non,f}$	0.381	0.464	0.462	0.519

^a^Each scenario included 1000 independent nominal sire–dam pairs, with 100 daughters simulated per family. Scenario labels indicate the dam pathway followed by the sire pathway: DD, DI, ID and II

At the within-scenario level, *V*_*f*_ was positively associated with observed family variance, although the strength of this association varied among scenarios. The *R*^*2*^ values were 0.037 in II, 0.089 in ID, 0.096 in DI, and 0.257 in DD. Therefore, the block-weighted segregation potential index captured the scenario-level ordering of family variance but explained only part of the variation among families within each scenario. This indicates that the index summarizes the segregation potential of each parental pair but does not fully determine the exact variance observed among the 100 simulated offspring, which also depends on stochastic recombination and Mendelian sampling.

To inform the subsequent heuristic sire-allocation procedure, we evaluated whether three readily computable descriptors of parental ancestry architecture were associated with family-level non-nominal risk: the distance of the expected family mean from the target, *d*_*f*_ = ∣*µ*_*arch, f*_ – p^*^∣, and the block-weighted segregation potential terms *V*_*f,1/8*_ and *V*_*f,1/16*_. We fitted the exploratory quasibinomial model in Eq. (18) using the number of non-nominal offspring out of 100 simulated daughters per family as the response (see [Additional file 4, Figure S4], [Additional file 5, Figure S5], [Additional file 6, Figure S6], and [Additional file 7, Figure S7] for diagnostic plots). This model was intended as a first-order screening model to identify covariates for the heuristic search, not as a complete generative model of offspring segregation.

Across all scenarios, distance to target was consistently associated with non-nominal risk (Table 3). The *V*_*f,1/8*_ component was also positively associated with risk in every scenario, whereas the *V*_*f,1/16*_ component showed a weaker and more scenario-dependent effect. Thus, family-level risk was associated with both the centring of the expected offspring distribution and the segregation structure of the parental genomes.

**Table 3:** Scenario-specific quasibinomial models of family-level non-nominal risk

Scenario	Intercept	d_f_	V_f,1/8_	V_f,1/16_	*R* ^2^	Dispersion
DD	-1.232 (< 2 × 10^− 16^)	26.069 (< 2 × 10^− 16^)	672.898 (< 2 × 10^− 16^)	704.293 (1.49 × 10^− 10^)	0.374	1.103
DI	-0.545 (6.18 × 10^− 16^)	16.679 (< 2 × 10^− 16^)	256.185 (1.84 × 10^− 8^)	149.070 (0.094)	0.208	1.008
ID	-0.676 (< 2 × 10^− 16^)	16.467 (< 2 × 10^− 16^)	293.869 (1.61 × 10^− 9^)	356.547 (2.43 × 10^− 4^)	0.196	1.055
II	-0.225 (0.0066)	10.462 (< 2 × 10^− 16^)	139.169 (2.00 × 10^− 4^)	-12.569 (0.898)	0.096	1.033

Coefficients are shown on the logit scale, with p-values in parentheses. R^2^ corresponds to explained-deviance pseudo-R^2^, calculated as 1 − residual deviance/null deviance. Dispersion values correspond to the quasibinomial model (Eq. (19))

This joint effect is shown by the predicted risk surfaces (Fig. 6). Across scenarios, predicted non-nominal risk increased as the expected family mean moved away from the target and as the block-weighted segregation potential index increased. However, the scenarios differed in their accessible risk space. II matings occupied a consistently high-risk region, whereas DD matings spanned a broader risk range and included substantially lower-risk parental combinations. ID and DI showed intermediate and broadly symmetric risk surfaces.

**Fig. 6:**
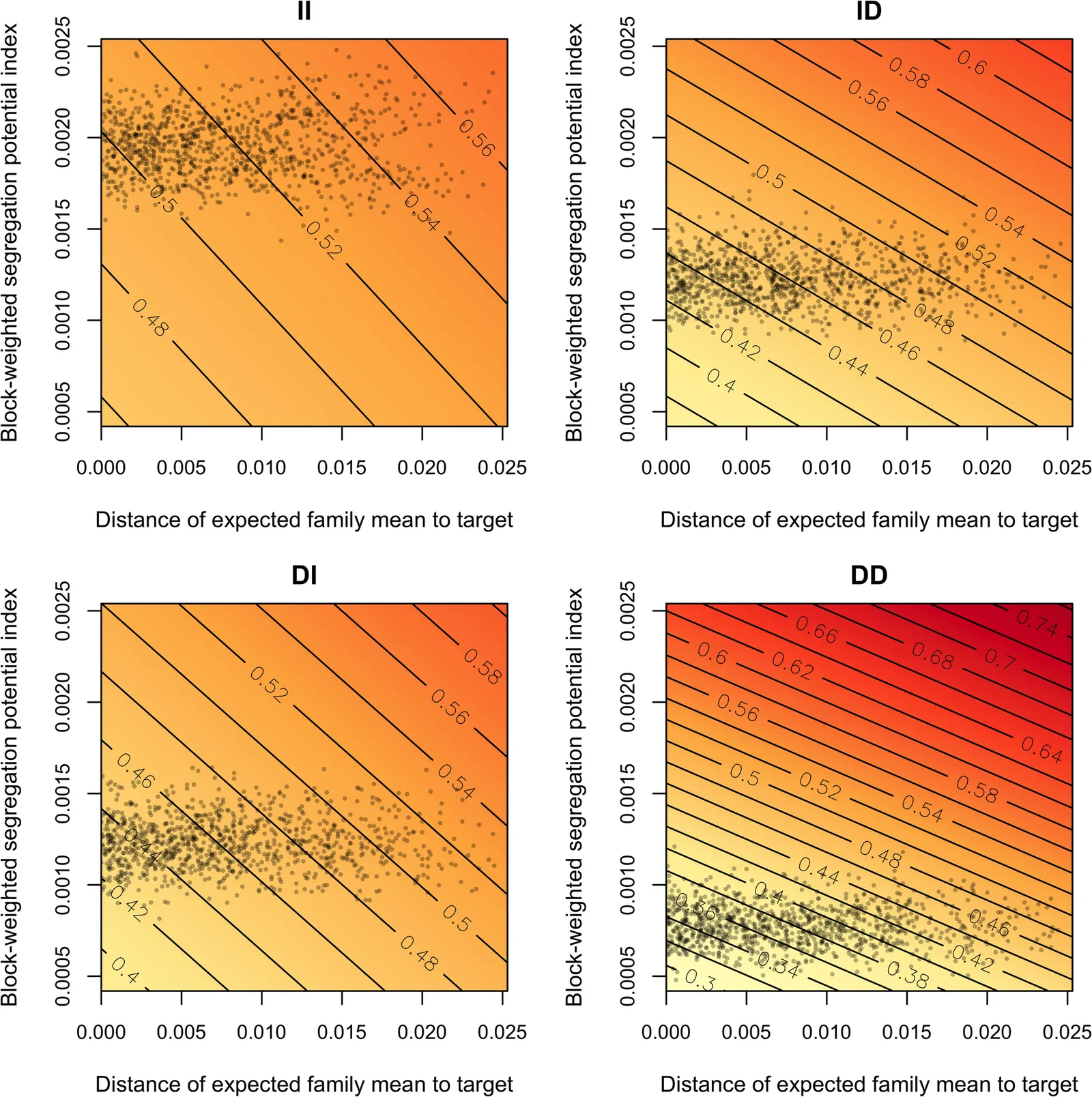
Predicted risk surfaces for non-nominal daughters across controlled nominal-parent mating scenarios. Panels represent dam–sire pathway combinations: D, direct; I, indirect. The x-axis shows the distance between the expected family mean Brahman ancestry and the target of 0.375; the y-axis shows the block-weighted segregation potential index. Background colours and contours indicate probabilities fitted by the scenario-specific quasibinomial models. Dark points represent sire–dam pairs, with 100 daughters simulated per pair. Axis ranges and colour scales are shared across panels

An extended exploratory model including interactions between *d*_*f*_ and the segregation-potential terms produced only small increases in explained deviance relative to the additive model across scenarios. Therefore, the additive model was retained as the main exploratory summary because it provided a simpler first-order description of the architecture-derived covariates used to inform the heuristic search.

Together, these results show that the nominal status of individual parents is insufficient to predict reproductive outcome. Even among nominal parents, family-level risk depends on the expected position of the cross relative to the target and on the block-weighted segregation structure of the parental genomes. Therefore, the relevant unit of control in composite-breed management is not only the individual animal, but the specific mating.

### Using mating-specific risk to improve sire allocation

Because empirical Brangus populations can deviate from the expected 3/8 Brahman composition, we next evaluated whether mating-specific genomic information could improve sire allocation when both dams and candidate sires vary around the target.

As a first approximation to this problem, we evaluated a simple sire-allocation strategy in a mixed commercial population. A fixed set of 1000 commercial dams was sampled from the direct and indirect female populations without filtering on nominal status. Sires were sampled from the full pool of 5000 direct-derived and 5000 indirect-derived candidate sires to create three catalogues of 100 sires each: a strict catalogue within 0.375 ± 0.025, a moderate catalogue within 0.375 ± 0.050, and a broad catalogue within 0.375 ± 0.100. These catalogues were intended to represent increasing levels of deviation around the target Brahman ancestry.

For each catalogue, we compared random sire allocation with a heuristic-assisted simulation-informed strategy, according to which all 100 candidate sires were first ranked for each dam using a heuristic based on the distance between the expected family mean and the target Brahman ancestry, *d*_*f*_, together with the block-weighted segregation potential index, *V*_*f*_. The 10 best-ranked sires were then evaluated by simulating 50 probe offspring per candidate mating. The sire with the lowest empirical non-nominal risk was selected, and one final independent offspring was generated from the selected mating.

Under random sire allocation, broadening the sire catalogue worsened nominal offspring yield. Hence, the proportion of nominal offspring decreased from 0.495 in the strict catalogue to 0.482 in the moderate catalogue and 0.451 in the broad catalogue (Table 4). In contrast, heuristic-assisted simulation-informed allocation increased nominal offspring yield in all catalogues, with the largest gains observed when broader sire catalogues were available. The proportion of nominal offspring increased from 0.554 in the strict catalogue to 0.611 in the moderate catalogue and 0.620 in the broad catalogue. Relative to random allocation within each catalogue, this corresponded to 59, 129 and 169 additional nominal offspring per 1000 matings, respectively.

The improvement was accompanied by reduced dispersion in offspring ancestry. Under random allocation, the standard deviation of offspring Brahman ancestry increased from 0.0365 in the strict catalogue to 0.0420 in the broad catalogue. Under heuristic-assisted simulation-informed allocation, the corresponding standard deviations were lower and more stable, ranging from 0.0309 to 0.0296. Thus, broader sire catalogues increased risk when used randomly but provided additional opportunity for compensatory matings when sire choice was informed by the expected outcome of each specific cross.

The improved nominal offspring yield was associated with less even sire use. Random allocation used all 100 sires in each catalogue, with a maximum of 18–22 offspring assigned to any single sire. In contrast, heuristic-assisted simulation-informed allocation used 41, 42 and 51 sires in the strict, moderate and broad catalogues, respectively, with maximum sire use ranging from 70 to 79 offspring. This indicates that the present strategy improved ancestry outcomes by concentrating matings on a subset of sires that were more favourable for the specific dam population.

The analysis should be interpreted as a proof of concept rather than as a final breeding optimization algorithm. Nevertheless, it demonstrates that mating-specific genomic information can be used to convert variation in sire ancestry from a source of risk into an opportunity for compensatory mating. Future implementations could incorporate constraints on sire use, inbreeding, genetic diversity, genetic merit and other breeding objectives, while retaining the central principle that the expected outcome of the sire–dam pair is more informative than the nominal status of either parent alone.

**Table 4:** Sire allocation outcomes under alternative sire catalogue ancestry intervals

Statistic	Strict catalogue		Moderate catalogue		Broad catalogue	
	Random	Informed	Random	Informed	Random	Informed
Sire ancestry interval	0.375 ± 0.025	0.375 ± 0.025	0.375 ± 0.050	0.375 ± 0.050	0.375 ± 0.100	0.375 ± 0.100
Number of daughters	1000	1000	1000	1000	1000	1000
Mean daughter ancestry	0.374	0.374	0.377	0.375	0.372	0.374
SD of daughter ancestry	0.037	0.031	0.039	0.030	0.042	0.030
Nominal daughters	495	554	482	611	451	620
Non-nominal daughters	505	446	518	389	549	380
Proportion nominal	0.495	0.554	0.482	0.611	0.451	0.620
Proportion non-nominal	0.505	0.446	0.518	0.389	0.549	0.380
Informed – Random (number)	--	+ 59	--	+ 129	--	+ 169
Informed – Random (percentage points)	--	+ 5.9%	--	+ 12.9%	--	+ 16.9%
Unique sires used	100	41	100	42	100	51
Maximum sire use	22	70	18	78	18	79

## Discussion

Our study shows that nominal Brangus ancestry does not define a unique genomic state, nor does it fully predict the reproductive outcome of an animal when used in a specific mating. Across the simulations, animals and sire–dam pairs could be equivalent in global Brahman ancestry proportion while differing in ancestry heterozygosity, homozygous ancestry composition, parental joint-state block structure and the probability of producing non-nominal offspring. The mixed *inter se* analysis further showed that genome-wide ancestry variance does not immediately collapse to the independent-locus expectation after composite formation. SD(*p*) declined gradually over 100 generations, consistent with progressive recombination of linked ancestry blocks, but remained above the idealised independent-locus reference over the simulated time horizon. This is relevant for practical Brangus management because many commercial or developing composite populations are unlikely to correspond to closed populations maintained for many generations without further founder-breed input. Instead, upgrading, recurrent use of Angus- or Brahman-influenced animals, and herd renewal may repeatedly introduce ancestry segments of different ages. Such populations may therefore resemble early-to-intermediate phases of the simulated mixed *inter se* process more than a long-term independent-locus limit. This is the type of setting in which mating-specific evaluation may be most useful, because nominal parental status alone may not reliably predict the distribution of offspring ancestry. Thus, the nominal 3/8 Brahman composition should be interpreted as a global ancestry descriptor, not as a complete description of genomic architecture or segregation behaviour.

Previous genomic studies in Brangus have shown that realised breed composition can deviate from the theoretical 3/8 Brahman and 5/8 Angus expectation, and that selection, drift and complementarity can shape the genomic architecture of composite populations [8, 9, 11, 12]. Our results are consistent with these observations but extend them in a different direction. Rather than asking only what the breed composition of an animal, population or genomic region is, we asked what distribution of breed composition is expected from a specific sire–dam pair. This shift is important because the practical objective in composite-breed management is not only to identify animals with acceptable ancestry composition, but also to design matings that maintain the desired composition in the next generation.

The individual-level simulations showed that two routes to the same expected Brahman ancestry can produce different distributions of ancestry composition and ancestry heterozygosity. Both the direct and indirect pathways achieved the nominal target at the level of the population mean, but they generated different individual distributions and different genomic architectures. The decomposition of nominal genomes into Angus homozygous, heterozygous and Brahman homozygous components clarified this point: the target value of 0.375 does not correspond to a single architecture, but to a continuum of possible genomic configurations. Consequently, filtering candidate sires into the nominal interval standardized their global Brahman ancestry but did not erase the structural footprint of the formation pathway.

This distinction is relevant because commercial herds are not composed only of filtered, nominal animals. In the herd simulations, mean Brahman ancestry remained close to the target across renewal scenarios, yet a large proportion of individual females remained outside the nominal interval. Therefore, monitoring breed composition only through population averages can be misleading. A herd can appear correctly centred while still retaining many individuals that deviate from the accepted range. This supports the need to evaluate both individual ancestry distributions and the genomic architecture of the parents used for renewal.

The controlled family-level analysis showed why nominal sires and dams can still produce non-nominal offspring. Even when both parents were required to be nominal, family-level risk differed markedly among mating scenarios. These differences were not primarily explained by shifts in the expected family mean, which remained close to the target across scenarios. Instead, the major difference was the dispersion of offspring ancestry around that expected mean. A mating may therefore be well centred on average and still produce many non-nominal offspring if its segregation variance is high. Conversely, another mating with a similar expected mean may produce a tighter offspring distribution and a lower non-nominal risk.

The parental joint-state block analysis provides a mechanistic interpretation of this segregation variance. Offspring do not inherit ancestry states as independent loci, but as chromosomal segments transmitted through meiosis. Thus, the arrangement of parental genotype states along the genome affects the variance of offspring ancestry. Blocks in which both parents are heterozygous generate the highest expected segregation variance, single-heterozygote blocks generate intermediate variance, and fully homozygous combinations generate no segregation variance. Because block contributions are weighted by the square of genomic length (Eq. (15)), long high-variance blocks have a disproportionate effect on the block-weighted segregation potential index. This framework links local parental architecture to family-level ancestry risk and complements previous genomic studies that have described breed composition, local ancestry and selection signatures in Brangus and other composite breeds [8, 9, 13, 14].

These findings connect with previous theoretical work in three specific ways. First, the comparison between the direct and indirect pathways showed that the same expected Brahman ancestry proportion can generate different realised individual ancestry distributions. This parallels the broader principle that the expected value of a genome-wide fraction may not fully describe the realised distribution of that fraction among individuals [23]. Second, filtering sires into the same nominal *p*_*i*_ interval did not remove pathway-dependent differences in ancestry heterozygosity and block structure. This is consistent with theory showing that the number and length of ancestral chromosome segments depend on population history and accumulated recombination [24]. Third, the mixed *inter se* analysis showed that SD(*p*) declined gradually over 100 generations rather than immediately reaching the independent-locus expectation. This agrees with local-ancestry models in which ancestry-proportion variance declines as recombination progressively increases the number of approximately independent ancestry units, while admixture timing, migration and drift can also affect tract-length distributions and ancestry variance [25].

The exploratory quasibinomial model (Eq. (18)) supported the same conclusion from an association-screening perspective. Family-level non-nominal risk was associated with both the distance between the expected family mean and the target, and with the block-weighted segregation potential components. These two terms represent different dimensions of risk: centring and spread. A cross can be risky because its expected mean is displaced from the target, because it has high segregation potential, or because both occur simultaneously. This distinction converts breed-composition management from a purely descriptive problem into a mating-specific evaluation problem.

The sire-allocation analysis illustrated the applied value of this framework. When sires were allocated randomly, broadening the sire catalogue worsened nominal daughter yield. In contrast, when sire choice was informed by mating-specific risk, broader catalogues provided additional opportunities to identify compensatory matings and increased the proportion of nominal daughters. This suggests that animals that deviate from the target are not necessarily detrimental if they are used selectively. A compositionally deviating but genetically valuable sire may still be useful if mated to dams for which it produces a well-centred and sufficiently narrow offspring distribution. Thus, mating-specific risk evaluation may help distinguish animals that are individually non-nominal but reproductively useful from animals likely to transmit excessive ancestry deviation across many matings.

From an operational perspective, the sire-allocation analysis should be viewed as a proof of concept rather than as a final breeding optimization algorithm. The heuristic was deliberately simple, and the improved nominal offspring yield was associated with less even sire use. Practical implementations would need to include constraints on sire use, inbreeding, genetic diversity, genetic merit, reproductive logistics and economic objectives. This is consistent with broader optimal mating approaches, in which sire–dam combinations are evaluated jointly to balance multiple breeding objectives [20]. The present contribution is to show that ancestry risk can be incorporated as one component of such a mating-allocation framework.

Although this study focused on breed-composition risk rather than performance, the results may also have implications for the interpretation of retained heterosis in composite breeds. Classical expectation for retained heterozygosity in a composite population is based on founder-breed fractions. When breeds contribute unequally to the foundation, the proportion of F1 heterozygosity retained after *inter se* mating is proportional to $\:1-{\sum\:}_{i}{P}_{i}^{2}$, where *P*_*i*_ is the fraction of founder breed *i* [1, 3]. Under this expectation, a two-breed composite with 5/8 contribution from one breed and 3/8 from another retains 46.9% of F1 heterozygosity [1, 3]. In the present framework, animals with the same nominal Brahman ancestry can differ in the amount and spatial distribution of ancestry heterozygosity. Therefore, mating-specific genomic information could also be used to evaluate whether a given sire–dam pair is expected to maintain or erode ancestry heterozygosity in its offspring.

However, this application should be interpreted with caution, because retained heterozygosity should not be equated with retained heterosis itself [1–3, 19, 26]. Retained heterozygosity is a genomic expectation based on breed fractions, whereas true retained heterosis is a phenotypic quantity: the proportion of F1 heterotic advantage that remains in later generations relative to additive breed expectations. Experimental studies in advanced-generation beef composites have shown that retained heterosis can be equal to, greater than, or lower than expectations based on retained heterozygosity depending on the trait and breed combination [1, 2, 26]. Thus, breed composition and heterozygosity provide useful expectations, but they do not by themselves measure phenotypic heterosis. Testing whether these architectural differences translate into differences in retained heterosis would require phenotypic data and models that explicitly connect local ancestry, dominance, epistasis and performance. Still, our results show that nominal composition alone does not predict a unique level or genomic distribution of ancestry heterozygosity. This distinction is important because ancestry heterozygosity is sometimes used as a genomic proxy related to retained heterosis in composite populations. In that context, *p*_*i*_ and *H*_*i*_ should not be treated as interchangeable descriptors: animals with the same nominal ancestry proportion can differ substantially in ancestry heterozygosity and ancestry-block architecture.

The study has limitations arising from its controlled simulation design. Founder populations were represented as fixed alternative ancestry states, and the two formation pathways were simplified representations of Brangus development. Real populations would include more complex founder histories, recombination patterns, linkage disequilibrium, selection, drift, pedigree structure and uncertainty in local ancestry estimation. The block-weighted segregation potential index is not a formal analytical variance estimator; rather, it provides a heuristic summary of segregation potential, not an exact prediction of within-family ancestry variance. Similarly, the mixed *inter se* sensitivity analysis should not be interpreted as estimating a final equilibrium variance; rather, it describes the persistence and gradual decay of ancestry dispersion over a time horizon already longer than many practical composite-breed formation and early-maintenance programmes. The nominal interval was treated as a fixed management threshold, although acceptable ancestry ranges may vary among breed associations, markets and breeding objectives. These simplifications were intentional: the aim was not to reproduce every detail of real Brangus populations, but to isolate the principle that animals with the same global ancestry can differ in genomic architecture and in the offspring distributions they generate.

Future work should evaluate this framework using real Brangus genotype data, including both global and local ancestry estimates, and should test whether block-weighted segregation potential indexes remain informative under realistic linkage disequilibrium and recombination structures. The sire-allocation approach should also be integrated with genetic merit, inbreeding control, sire-use constraints and uncertainty in ancestry estimation, particularly when using marker panels of different densities. More broadly, the same logic could be extended to other composite breeds and structured crossbreeding systems in which ancestry composition affects breeding decisions, including stabilized Zebu–European composites such as Girolando, Braford, Santa Gertrudis and Beefmaster [10, 13–18], to mention a few. The framework may also be relevant to hybrid breeding systems in which differentiated strains or parental populations are crossed to exploit heterosis or combining ability. For instance, diallel crosses among red tilapia strains and common carp strains have been used to estimate heterosis, additive effects and family performance for growth and survival traits, generating multiple full-sib families per strain combination [27, 28]. In such systems, mating-specific evaluation of offspring outcomes could complement selection on breeding value by identifying crosses expected to generate favourable family distributions, particularly when heterosis potential, ancestry composition or the expected proportion of useful progeny is part of the breeding objective.

Overall, this study shows that maintaining nominal ancestry in a composite breed is not only a question of selecting animals that individually fall within a target interval. It also requires attention to how parental genomes combine and segregate in specific matings. Previous studies have shown that realised Brangus genomes can deviate from the theoretical breed composition [8, 11, 12]. Here, we show that such deviations can be partly understood and managed through mating-specific genomic information. In composite-breed management, nominal ancestry is a property of the individual; nominal offspring risk is a property of the mating.

## Conclusions

This study shows that the nominal 3/8 Brahman composition of Brangus animals should not be interpreted as a unique genomic state. Alternative breeding pathways can generate animals with similar global Brahman ancestry but different ancestry heterozygosity, homozygous ancestry composition and segregation structure. As a result, animals that are equivalent in nominal ancestry can differ in their probability of producing non-nominal offspring.

Population mean ancestry and individual nominal status were insufficient descriptors for managing breed composition. Commercial herds could remain close to the target ancestry on average while retaining a high proportion of non-nominal individuals, and matings between nominal parents differed in offspring risk depending on the expected family mean and the block-weighted segregation structure of the parental genomes. These results indicate that the relevant unit of control is not only the individual animal, but the specific sire–dam pair.

Mating-specific genomic information can be used to evaluate whether a cross is expected to produce an offspring distribution that is well centred on the target and sufficiently narrow to maintain a high proportion of nominal progeny. The proof-of-concept sire-allocation analysis showed that this information can improve nominal daughter yield relative to random sire use, especially when broader and more variable sire catalogues are available. These findings provide a conceptual and practical framework for managing breed composition in Brangus and potentially in other composite breeds or structured crossbreeding systems.

## Supplementary Information

Below is the link to the electronic supplementary material.

Supplementary Material 1

Supplementary Material 2

Supplementary Material 3

Supplementary Material 4

Supplementary Material 5

Supplementary Material 6

Supplementary Material 7

## Data Availability

The simulation code and datasets generated and analysed during the current study are available from the corresponding author on reasonable request.
